# Secondhand Smoke Decreased Excitability and Altered Action Potential Characteristics of Cardiac Vagal Neurons in Mice

**DOI:** 10.3389/fphys.2021.727000

**Published:** 2021-09-24

**Authors:** Junqing Sun, Shiyue Pan, Emma Karey, Yi-Je Chen, Kent E. Pinkerton, Christopher G. Wilson, Chao-Yin Chen

**Affiliations:** ^1^Department of Pharmacology, University of California, Davis, Davis, CA, United States; ^2^Department of Pediatrics and Center for Health and the Environment, University of California, Davis, Davis, CA, United States; ^3^Department of Basic Sciences, Loma Linda University, Loma Linda, CA, United States

**Keywords:** environmental tobacco smoke, autonomic function, nucleus ambiguus, SK channel, neuroplasticity, spiking activity, cardiovascular

## Abstract

**Background:** Secondhand smoke (SHS), a major indoor pollutant, is a significant risk factor for cardiovascular morbidity and mortality including arrhythmias and sudden cardiac death. Exposure to SHS can produce autonomic imbalance, as evidenced by reduced heart rate variability (HRV)—a clinical metric of cardiac vagal regulation. Currently, the mechanisms through which SHS changes the vagal preganglionic neuronal inputs to the heart to produce this remains unknown.

**Objectives:** To characterize the effect of SHS on both the excitability and action potential (AP) characteristics of anatomically identified cardiac vagal neurons (CVNs) in the nucleus ambiguus and examine whether SHS alters small conductance calcium-activated potassium (SK) channel activity of these CVNs.

**Methods:** Adult male mice were exposed to four weeks of filtered air or SHS (3 mg/m^3^) 6 h/day, 5 day/week. Using patch-clamp recordings on identified CVNs in brainstem slices, we determined neuronal excitability and AP characteristics with depolarizing step- and ramp-current injections.

**Results:** Four weeks of SHS exposure reduced spiking responses to depolarizing current injections and increased AP voltage threshold in CVNs. Perfusion with apamin (20 nM) magnified these SHS-induced effects, suggesting reduced SK channel activity may serve to minimize the SHS-induced decreases in CVNs excitability. Medium afterhyperpolarization (a measurement of SK channel activity) was smaller in the SHS group, further supporting a lower SK channel activity. AP amplitude, rise rate, fast afterhyperpolarization amplitude (a measurement of voltage-gated channel activity), and decay rate were higher in the SHS group at membrane voltages more positive to 0 mV, suggesting altered inactivation properties of voltage-dependent channels underlying APs.

**Discussion:** SHS exposure reduced neuronal excitability of CVNs with compensatory attenuation of SK channel activity and altered AP characteristics. Neuroplasticity of CVNs could blunt regulatory cardiac vagal signaling and contribute to the cardiovascular consequences associated with SHS exposure, including reduced HRV.

## Introduction

The cardiovascular system is highly sensitive to the adverse effects of secondhand smoke (SHS), which contains more than 7,000 chemicals, including hundreds of known toxicants (Moritsugu, 2007; Warren et al., 2014). SHS exposure-related increases in cardiovascular morbidity and mortality have been shown to be as large as 80–90% of those from chronic active smoking (Barnoya and Glantz, 2005) and are believed to share many of the same underlying mechanisms as active smoking (Moritsugu, 2007; Warren et al., 2014). Three broad, non-mutually exclusive, pathways have been proposed to mediate the cardiovascular detriments attributed to SHS: (1) systemic spill-over of inflammatory and/ or oxidative stress mediators into the circulation, (2) autonomic imbalance favoring sympathetic activation, and (3) penetration of particulates into cardiovascular tissues (Franklin et al., 2015).

SHS exposure-induced autonomic imbalance, as measured by reduced heart rate variability (HRV), may be particularly important because a shift in autonomic regulation can trigger acute cardiovascular events (e.g., arrhythmias and sudden cardiac death) as well as contribute to the development of chronic cardiovascular disease states (e.g., hypertension and vascular hypertrophy) (Franklin et al., 2015). Although HRV is a valuable biomarker of autonomic regulation (Task_Force, 1996), relative contributions from cardiac vagal vs. sympathetic inputs that manifest as SHS-mediated attenuated HRV are poorly characterized. Direct measurement of muscle sympathetic nerve activity (SNA) showed increased muscle SNA in non-smokers exposed to SHS, which has been interpreted as evidence of increased cardiac SNA after SHS exposure (Hausberg et al., 1997). However, direct measurement of cardiac SNA in a rabbit animal model found that cigarette smoke exposure induced a fall in cardiac SNA while increasing renal SNA, suggesting that differential patterns of sympathetic outflow can be elicited by smoke exposure (Peterson et al., 1983).

For the parasympathetic limb of cardiac autonomic regulation, obtaining direct measurement of changes in neuronal activity or vagal efferent activities has been challenging. Time domain HRV measures (e.g., root mean square of successive difference) and frequency domain HRV (i.e., high frequency band power) have been used as surrogate indices of vagal regulation (Task_Force, 1996; Moritsugu, 2007; Warren et al., 2014; Franklin et al., 2015). Several studies have demonstrated an association between SHS exposure and decreased HRV, both acutely and in chronic exposure settings (Pope et al., 2001; Barnoya and Glantz, 2005; Chen et al., 2008; Wilson et al., 2010; Zhang et al., 2013). However, we lack direct mechanistic evidence of how SHS exposure changes cardiac vagal nerve activities that result in the phenotypic shift in autonomic balance that have been previously described.

Cardiac vagal neurons (CVNs) in the *nucleus ambiguus* (NA) have been shown to play a key role in regulation of heart rate (Corbett et al., 1999; Mendelowitz, 1999; Cheng et al., 2004). These CVNs send projection to the heart and are silent at resting membrane potential (Mendelowitz, 1996; Pham et al., 2009). Ongoing tonic and phasic dynamic vagal activity to the heart are determined by the integration of intrinsic excitability on excitatory depolarizing inputs. Changes in intrinsic excitability of CVNs can mute or amplify synaptic inputs to shape the final output of these cells that are responsible for cardiac regulation. In the central nervous system small conductance calcium-activated potassium channel (SK) channels have been shown to play an important role in finetuning action potential discharge frequency during repetitive firing and in synaptic integration (Bond et al., 2005). The CVNs also express SK channels (Lin et al., 2010a,b, 2011, 2014). In CVNs, these SK channels have been shown to be activated during repetitive firing and shape the final neuronal output (Mendelowitz, 1996; Lin et al., 2010a,b). The objective of this study was to characterize the effect of SHS on both the excitability and action potential (AP) characteristics of anatomically identified CVNs in the nucleus ambiguus and examine whether SHS alters SK channel activity of these CVNs. We hypothesize that SHS exposure reduces intrinsic excitability of CVNs by increasing SK channel activity.

## Materials and Methods

All experimental protocols were approved by the Institutional Animal Care and Use Committee at the University of California, Davis and in compliance with the Animal Welfare Act (Office of Laboratory Animal Welfare 2002) and Public Health Service Policy on Humane Care and Use of Laboratory Animals (Animal Welfare Act 1966). All mice were treated humanely and with regard for the alleviation of suffering, consistent with the guidelines provided by the National Institutes of Health. All mice were housed in a 12-h light/ 12-h dark cycle with *ad libitum* access to regular rodent chow and water. The housing temperature was 21 ± 3°C (mean ± SD) and relative humidity was 67 ± 18% (mean ± SD).

### Retrograde Labeling of CVNs With a Fluorescent Tracer

All surgeries were performed under sterile conditions. Seven- to eight-week old male C57BL/6J mice (The Jackson Laboratory, CA, USA) were anesthetized with isoflurane (1.5–5% in 100% oxygen). As is routinely done in our laboratory (Pham et al., 2009), we performed a right thoracotomy and placed a parafilm patch (1 mm^2^) coated with fluorescent tracer 1,1'-dioctadecyl 3,3,3',3'-tetramethylindo-carbocyanine perchlorate (DiI) over the sinoatrial node region. The parafilm patch was secured and sealed with tissue glue. Lungs were slightly hyperinflated before the closure of the chest. All mice received buprenex (0.05 mg/kg) for preemptive analgesia and twice daily for two post-op days.

### SHS Exposure

Two weeks after the surgery, mice were randomly assigned to be either exposed to filtered air (FA) or to SHS for four weeks (6 h/day, 5 days/week). As in our previous studies (Chen et al., 2008; Sekizawa et al., 2008; Wang et al., 2018), sidestream cigarette smoke, a surrogate for secondhand smoke, was generated from conditioned 3R4F research cigarettes using a modified ADL/II system (Little Cambridge, MA, USA). The 3R4F cigarette, an international standard reference cigarette for smoke research (Hamad et al., 2017), was obtained from the University of Kentucky Tobacco and Health Research Institute (Lexington, KY, USA) and smoked under Federal Trade Commission conditions (2-second puff of 35 cm^3^ volume, one puff per min). Smoke was collected and diluted with FA in a mixing chamber to achieve the target total suspended particulate (TSP) concentration of 3 mg/m^3^, and then delivered to a 0.44 m^3^ glass Hinners-type exposure chamber where the mice were exposed (whole body exposure) in their home cage with wire lids, rodent chow, and water *ad libitum*.

The averaged real-life fine particulate matter concentration in smoking restaurants and bars has been reported to be 0.2–0.6 mg/m^3^ with the maximal level reaching 3 mg/m^3^ (Semple et al., 2007; Liu et al., 2011; Pacheco et al., 2012). We chose the high end of realistic exposure level (3 mg/m^3^ of TSP) for this study. Exposures were characterized daily for three major components of cigarette smoke (means ± SD): nicotine (0.3 ± 0.2 mg/m^3^), carbon monoxide (14.3 ± 1.1 ppm), and TSP (3.0 ± 0.1 mg/m^3^).

### Slice Preparation

After four weeks of SHS or FA exposure, mice (now 13–14 weeks old) were anesthetized with 5% isoflurane and decapitated. The head was immediately submerged for 30 s in a slush made from high-sucrose artificial cerebrospinal fluid (aCSF) that contained (in mM) 73 NaCl, 2.5 KCl, 2 MgCl_2_, 1.25 NaH_2_PO4, 25 NaHCO_3_, 24 dextrose, 0.5 CaCl_2_ and 75 sucrose (300 mOSM, continuously bubbled with 95%O_2_-5%CO_2_). The brain was then exposed and submerged in ice-cold high-sucrose aCSF. Transverse brainstem slices (150 μm) were cut with a Leica VT1000S vibrating microtome (Leica microsystems, Inc. Bannockburn, IL). After incubation at 35°C for 45 min in high-sucrose aCSF, slices were placed in regular aCSF that contained (mM) 128 NaCl, 2.5 KCl, 1 MgCl_2_, 1.25 NaH_2_PO4, 25 NaHCO_3_, 10 dextrose and 2 CaCl_2_ (300 mOSM, continuously bubbled with 95%O_2_-5%CO_2_).

### Whole-Cell Patch-Clamp Recordings

**Figure 1:**
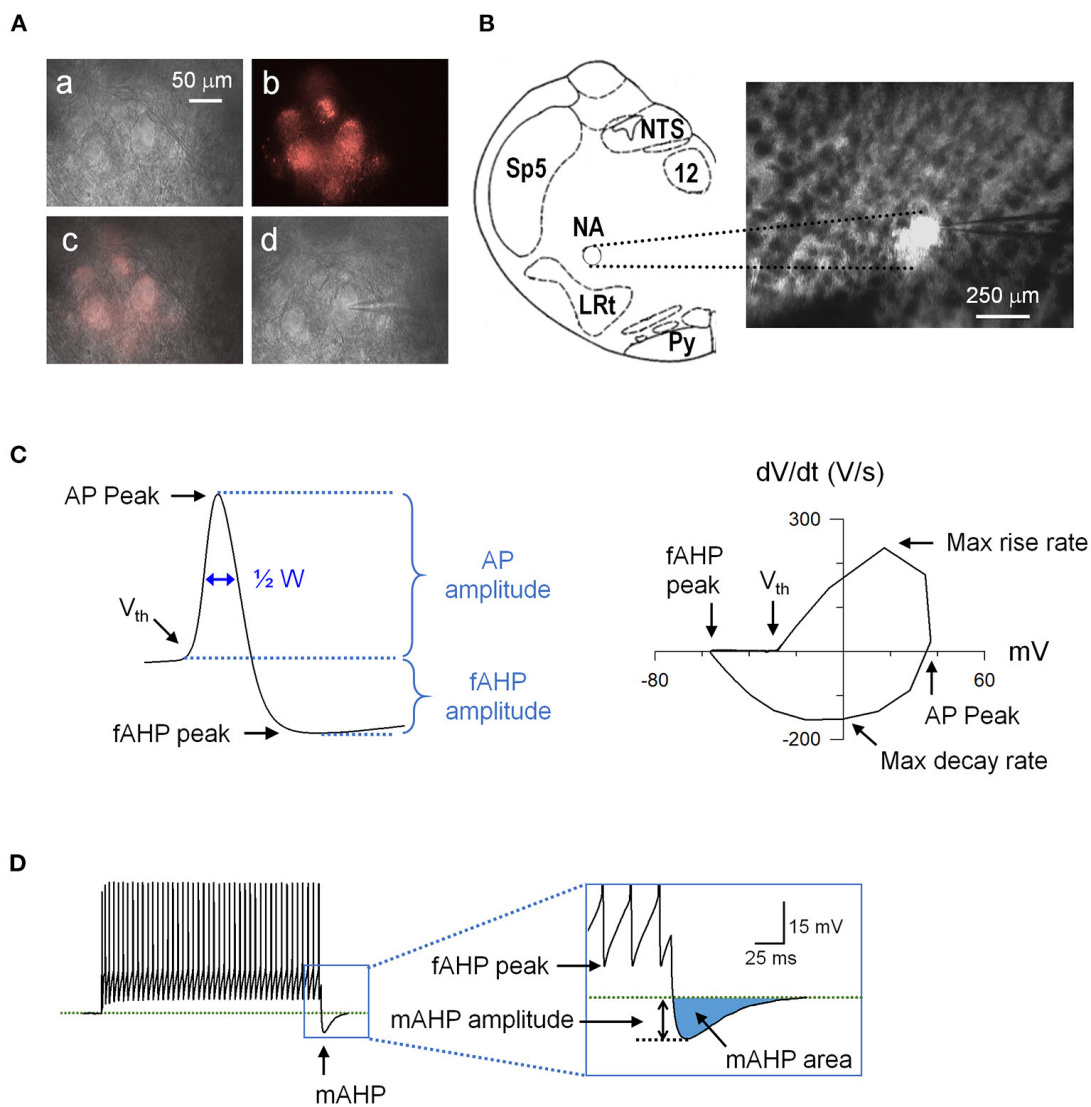
An example of retrogradely labeled cardiac vagal neurons (CVNs) and action potential (AP) analysis. **(A) (a)** Nucleus ambiguus region viewed at 40x with Infrared-differential interference contrast (IR-DIC). **(b)** The same region viewed with a fluorescence filter for DiI. **(c)** Overlay of the IR-DIC and fluorescence images. **(d)** An identified CVN with a patch electrode in whole-cell configuration. **(B)** Schematic drawing of the recording site (left) and the brainstem slice containing the nucleus ambiguus viewed at 5x. LRt, lateral reticular nucleus; NA, nucleus ambiguus; NTS, nucleus tractus solitarii; Py, pyramidal tract; Sp5, spinal trigeminal nucleus; 12, hypoglossal nucleus. **(C)** A recorded action potential (left) and its phase plane plot (right) showing the measured parameters. **(D)** Spiking response to a 1-s depolarizing current step showing the mAHP. AHP, afterhyperpolarization; ½ W, AP half width.

For recordings, a single slice was placed in the recording chamber, held in place with a silk meshed platinum ring, and continuously perfused with oxygenated aCSF at a rate of 4 ml/min. Whole-cell patch-clamp recordings were performed at 33–34°C on fluorescently labeled CVNs in the NA (Figures 1A,B). Neurons were identified with infrared differential interference contrast (IR-DIC), and the fluorescence was detected with an optical filter set for DiI (XF 108, Omega Optical Inc., Brattleboro, VT). Recording electrodes (2–5 MΩ) were pulled from a borosilicate glass capillary and filled with solution containing (in mM) 10 KCl, 128 K-gluconate, 2 MgCl_2_, 10 HEPES, 5.5 EGTA, 2 Na_2_-ATP and 0.1 CaCl_2_. Current clamp recordings were made with a MultiClamp 700B amplifier (Axon instruments, Sunnyvale, CA), filtered at 2 kHz, and digitized at 10 kHz with a Digidata 1440A (Axon Instruments). All neurons had seal resistance > 1 GΩ and series resistance <20 MΩ.

Immediately after obtaining the whole-cell configuration, resting membrane potential was determined in *I* = 0 mode and the neuron was then current-clamped at −60 mV. Steady-state input resistance was determined with hyperpolarizing currents (20–80 pA, 200 ms). To determine the delay in spiking response to depolarization, each neuron was injected with a hyperpolarizing current step to set the membrane potential to −80 mV for 500 ms (to remove inactivation of voltage-gated channels), followed by a 600 pA depolarizing current step (Sekizawa et al., 2012). Neurons with a delay time <10 ms were classified as rapid-onset spiking phenotype (RS) and neurons with a delay time > 10 ms were classified as delayed-onset spiking phenotype (DS) (Supplementary Figure 1).

To determine the intrinsic excitability of spiking responses to depolarization and action potential (AP) characteristics of CVNs, we used two current injection protocols. First, one-second depolarizing current steps (100–700 pA in 100 pA increments) were used to test general input-output relationship. Second, a five-second ramp (1 nA/s) current injection was used to determine the voltage threshold for AP generation and AP characteristics at membrane voltages that induce inactivation of voltage-gated ion channels. We measured the following AP waveform parameters (Figure 1C): AP peak (the absolute peak voltage), AP amplitude (voltage difference between AP peak and voltage threshold), voltage threshold, AP half width, peak of intra-train afterhyperpolarization (fast afterhyperpolarization peak, fAHP peak: absolute fAHP voltage), fAHP amplitude (voltage difference between fAHP peak and voltage threshold), maximum rise rate, and maximum decay rate. In addition, post-train AHP (medium AHP, mAHP) was measured at the cessation of step current injections (Figure 1D). We determined mAHP amplitude (the voltage difference between mAHP peak and baseline membrane voltage) and mAHP area (the area of the undershoot after the post-train). To determine the contribution of the SK channels to SHS exposure-induced changes in intrinsic excitability, we repeated these protocols in the presence of apamin (20 nM), a selective SK channel blocker.

### Data Analysis

Data are expressed as mean ± SE unless indicated otherwise. All statistical analyses were performed with GraphPad Prism (GraphPad Software, Inc.). Membrane properties (resting membrane potential, cell capacitance, and whole-cell resistance) were compared with a *t*-test (FA vs. SHS). For the step current injection protocol, the input-output (injected current—total evoked spikes) relationship and AP characteristics were compared using a two-way repeated measures ANOVA (currents = within factor, exposure = between factor). The minimum current required to evoke an AP was compared with the Mann-Whitney Rank Sum Test. AP-to-AP changes in instantaneous frequency and AP characteristics were analyzed for the first 15 APs of two current steps (500 and 600 pA) and analyzed with a three-way repeated measures ANOVA (current and AP number = within factors, exposure = between factor). In addition, spiking responses were grouped by total number of discharged spikes (21–25 spikes and 31–35 spikes) and average membrane voltage during current steps [−30 mV (−29 to −31 mV) and −26 mV (−25 to −27 mV)]. Data were analyzed using a three-way repeated measures ANOVA (AP number = within factor, total spike/membrane voltage and exposure = between factors).

For the ramp current injection protocol, the voltage and current at which the first AP occurred (voltage threshold and current threshold, respectively) were compared with a *t*-test (FA vs. SHS). As was done for step current injection outcomes, data were plotted based on injected current (250 pA increments), membrane voltage (5 mV increments), and instantaneous frequency (10 Hz increments) and compared with a two-way repeated measures ANOVA (injected current/instantaneous frequency = within factor, exposure = between factor) or mixed-effects model (membrane voltage).

For effects of apamin on membrane excitability in the step current injection protocol, a three-way repeated measures ANOVA was used for comparison (exposure/total spikes/membrane voltage = between factor, apamin/current/AP number = within factors). For the ramp current protocol, spiking and membrane voltage response to the ramp currents were analyzed with a three-way repeated measures ANOVA (exposure = between factor, apamin and current/membrane voltage = within factors). For effects of apamin on AP characteristics, data were expressed as delta changes from pre-apamin perfusion and analyzed with a mixed-effects analysis. *p* < 0.05 was considered significant.

## Results

All results were obtained from fluorescently (DiI) labeled CVNs in the NA (Figure 1). There was no difference between FA and SHS in the distribution of RS vs. DS neurons (Supplementary Table 1), thus, RS and DS neurons were combined in the data analysis. There was also no difference in CVN membrane properties including resting membrane potential, cell capacitance, and whole cell membrane resistance (Supplementary Table 1).

### Spiking Response to Step Current Injections

**Figure 2:**
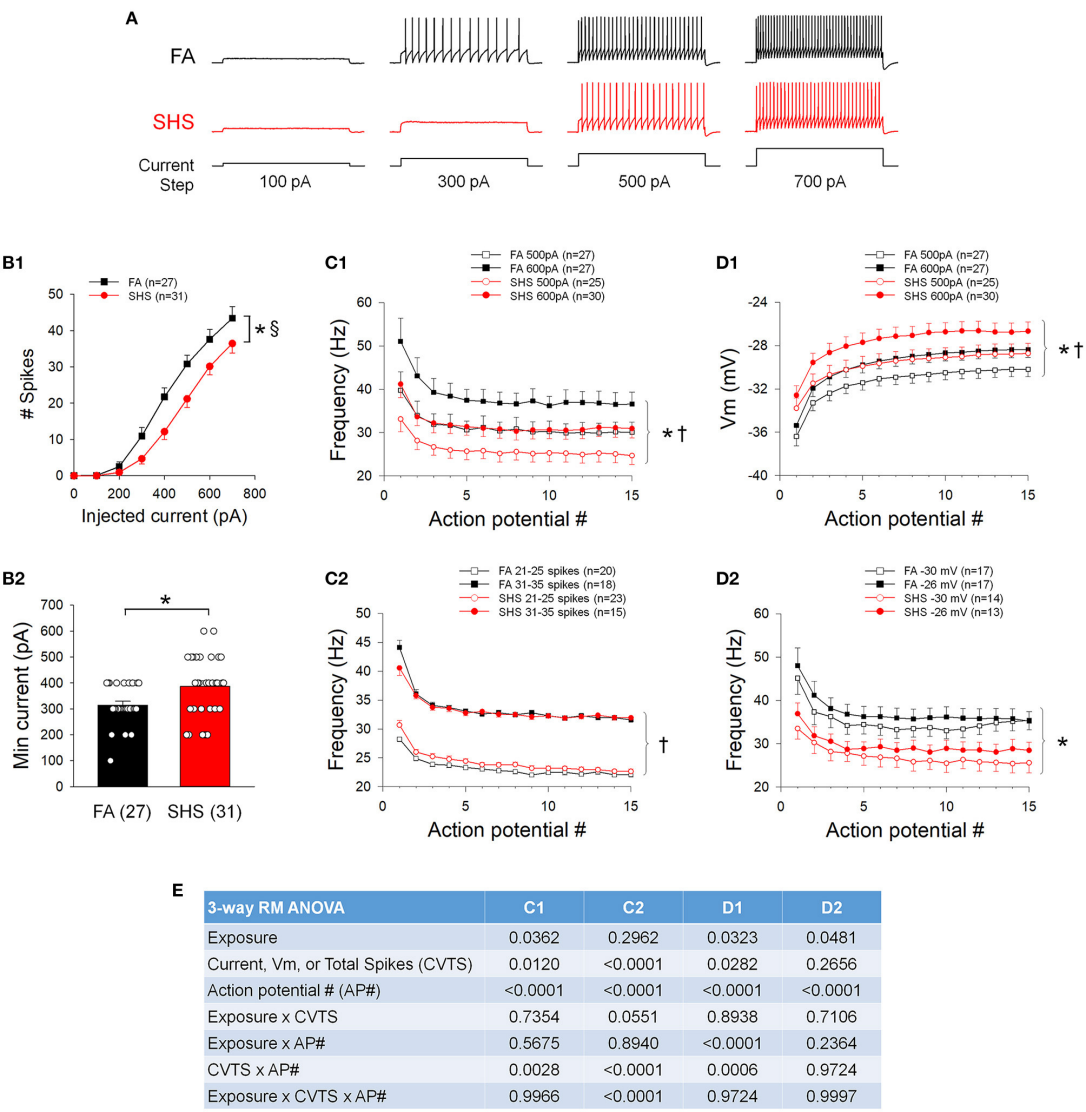
Effects of SHS on neuronal response to 1 s depolarizing current steps. **(A)** Example traces of depolarizing current injection induced spiking responses of CVNs from one filtered air (FA) and one secondhand smoke (SHS) exposed group. **(B1)** Total number of spikes discharged to 1 s depolarizing current steps. CVNs from the SHS exposed group had a lower spiking response compared to those from the FA group (two-way repeated measures ANOVA: *p* = 0.017 for exposure, *p* < 0.001 for injected current and *p* < 0.001 for interaction). **(B2)** Minimum current that evoked an AP was significantly higher in the SHS group (Mann-Whitney Rank Sum Test, *p* = 0.012). **(C1)** Instantaneous frequency of the first 15 APs at injected currents of 500 and 600 pA. Neurons from both groups showed similar frequency adaptation, albeit lower overall frequency in the SHS group compared to the FA control group. **(C2)** Instantaneous frequency of the first 15 APs from neurons discharged a total of 21–25 spikes and 31–35 spikes in response to 1 s depolarizing current steps, further demonstrate that there was no difference in frequency adaptation between FA and SHS exposed groups. **(D1)** Membrane voltage (Vm) at 500 and 600 pA current steps. Compared to the FA group, the membrane voltage was significantly more depolarized in the SHS group at each injected current, suggesting that the reduced spiking response in SHS group is not due to a more hyperpolarized membrane voltage at each injected current. **(D2)** Instantaneous frequency of the first 15 action potentials at two membrane voltages (−31 to −29 mV and −27 to −25 mV for −30 and −26 mV, respectively) demonstrating that the reduced spiking activity in SHS group persisted at similar membrane voltages. **(E)** Three-way repeated measures ANOVA results for panels **(C1,C2,D1,D2)**. *p < 0.05 SHS vs. FA, ^†^*p* < 0.05 main effects for current or total spikes, ^§^*p* < 0.05 exposure x current interaction.

Figure 2A shows examples of spiking responses to step current injections from one FA and one SHS neuron. As step current increased, neurons discharged more spikes (Figure 2B1). There was an overall exposure effect indicating that SHS mice discharged fewer total spikes. Additionally, a significant exposure by current interaction indicated that differences in spiking response between FA and SHS was greater at higher injected current (43 ± 3 and 36 ± 3 Hz at 700 pA, FA and SHS, respectively). This reduced spiking response was associated with a 23% increase in minimum current required to evoke an AP (Figure 2B2). Instantaneous frequency of the first 15 APs at current steps of 500 and 600 pA showed the highest instantaneous discharge frequency between the first and second APs (Figure 2C1). There was a slight decrease in instantaneous firing frequency over the first few APs, which ultimately stabilized within the first 10 APs. No difference (see ANOVA results in Figure 2E) in the frequency adaptation pattern was observed between FA and SHS neurons, although SHS neurons had an overall reduced discharge frequency compared to FA neurons injected with the same current.

To account for differences in overall number of spikes discharged at the same current steps, spiking responses were grouped by total discharged spikes recorded during 1 s current steps (Figure 2C2). Similar frequency adaptation was observed between FA and SHS groups. Because a higher depolarizing current was required to evoke an AP in the SHS group, we examined the potential impact of membrane voltage responses on overall spiking activity at current steps of 500 pA and 600 pA. The membrane voltages were significantly more depolarized in the SHS group at the same injected current (Figure 2D1). Thus, CVNs in the SHS group had reduced spiking response to current injections despite achieving a greater depolarizing membrane voltage from the same injected current. Spiking responses were further grouped based on the membrane voltage during 1 s current steps. Compared to the FA neurons, spiking activity was significantly blunted in SHS CVNs at the same membrane voltage (Figure 2D2).

### Step Current Injections and AP Waveform

**Figure 3:**
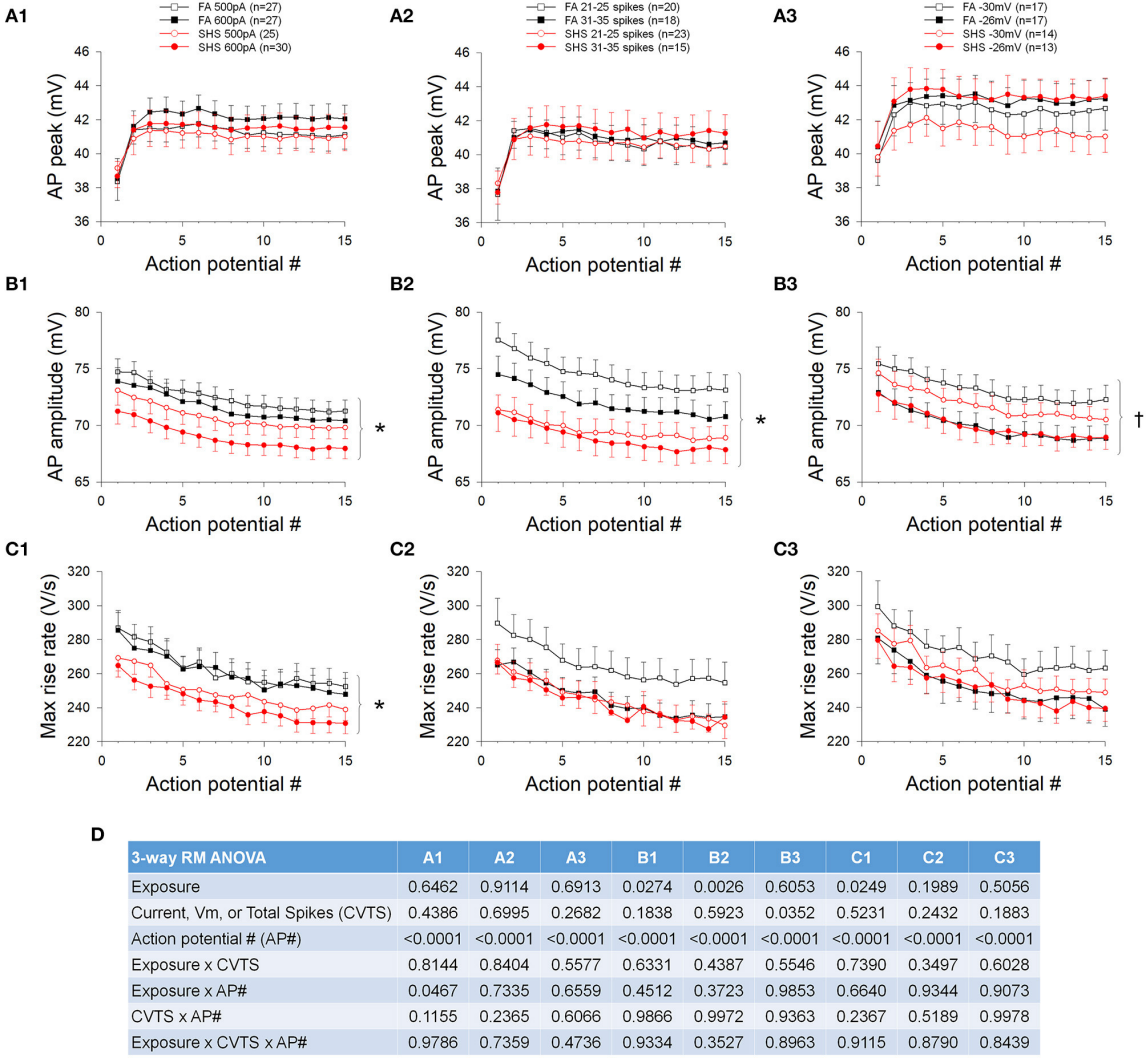
Effects of SHS on AP upstroke characteristics of the first 15 APs during step current injections. **(A)** Absolute AP peak voltage. There was no significant exposure effect on the AP peak whether grouping the data with same injected currents **(A1)**, total number of spikes **(A2)** or membrane voltages **(A3)**. **(B)** AP amplitude (measured from voltage threshold to AP peak). SHS exposure group had significantly lower AP amplitude when grouped with same injected currents **(B1)** or total number of spikes **(B2)** but not with same membrane voltages **(B3)**. **(C)** AP maximum rise rate. Compared to the FA group, the SHS group had a lower rise rate at the same injected current **(C1)**. There was no difference when grouping data by total number of spikes **(C2)** or membrane voltages **(C3)**. **(D)** Three-way repeated measures ANOVA results. CVTS, injected current, membrane voltage, or total spikes. *p < 0.05 SHS vs. FA, ^†^*p* < 0.05 main effect for membrane voltage.

The absolute AP peak voltage did not change with increasing depolarizing current, different spiking activities, membrane voltages, or SHS exposure (Figure 3A). AP amplitude, measured from voltage threshold to AP peak, was smaller in neurons from the SHS group at the same injected current (Figure 3B1) and total spike number (Figure 3B2). There was no significant exposure effect on AP amplitude when data were grouped by membrane voltage, however, AP amplitude was significantly smaller at more depolarized membrane voltage (Figure 3B3). The maximum rise rate of the AP upstroke at the same injected current was significantly lower in the SHS group (Figure 3C1). No exposure effect was detected when grouping the data by total spike number (Figure 3C2) or membrane voltage (Figure 3C3). ANOVA results are presented in Figure 3D.

**Figure 4:**
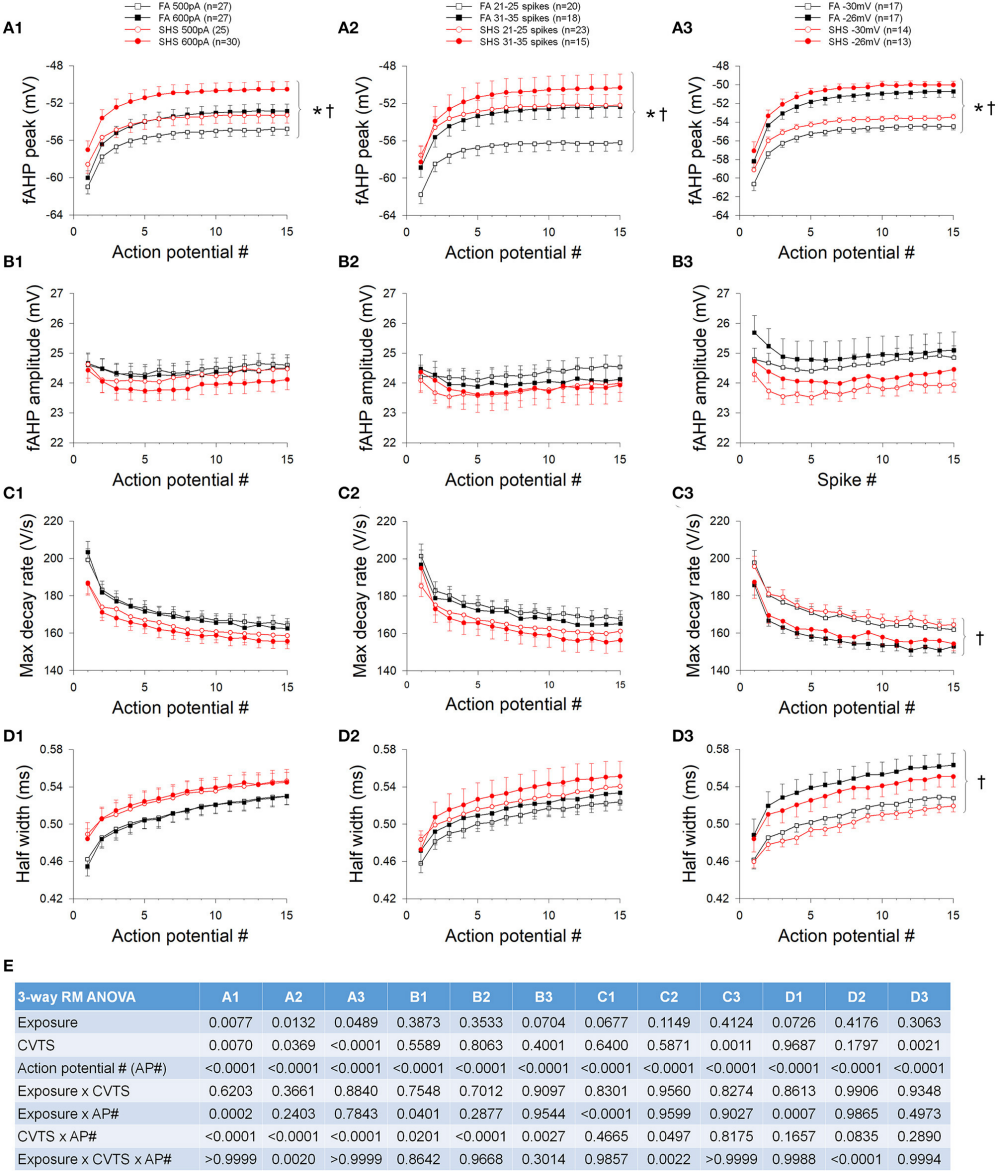
Effects of SHS on AP downstroke characteristics of the first 15 APs during step current injections. **(A)** Absolute fAHP peak voltage. The SHS group had more depolarized fAHP peak at same injected currents **(A1)**, total number of spikes **(A2)**, and membrane voltages **(A3)**. **(B)** fAHP amplitude (measured from voltage threshold to fAHP peak). SHS did not have significant effects on fAHP amplitude. **(C)** AP maximum decay rate showing no exposure effect whether the data were groups by injected current **(C1)**, total number of spikes **(C2)**, or membrane voltage **(C3)**. **(D)** AP half width showing no difference between FA and SHS whether grouping the data by injected current **(D1)**, total number of spikes **(D2)** or membrane voltage **(D3)**. **(E)** Three-way repeated measures ANOVA results. CVTS, injected current, membrane voltage, or total spikes. *p < 0.05 SHS vs. FA, ^†^*p* < 0.05 main effect for current, total spikes, or membrane voltage.

In contrast to the AP peak, absolute fAHP peak voltage was more depolarized in the SHS neurons regardless of whether data were grouped by injected current (Figure 4A1), discharge frequency (Figure 4A2), or membrane voltage (Figure 4A3). Furthermore, fAHP peak was more depolarized at higher injected current, discharge frequency, and more depolarized membrane voltage (Figure 4A). SHS exposure had no effect on fAHP amplitude (Figure 4B) maximum AP decay rate (Figure 4C), or AP half width (Figure 4D). Higher membrane voltage is associated with a lower maximum AP decay rate (Figure 4C3) and wider AP half width (Figure 4D3). ANOVA results are presented in Figure 4E.

### SHS Exposure and mAHP

**Figure 5:**
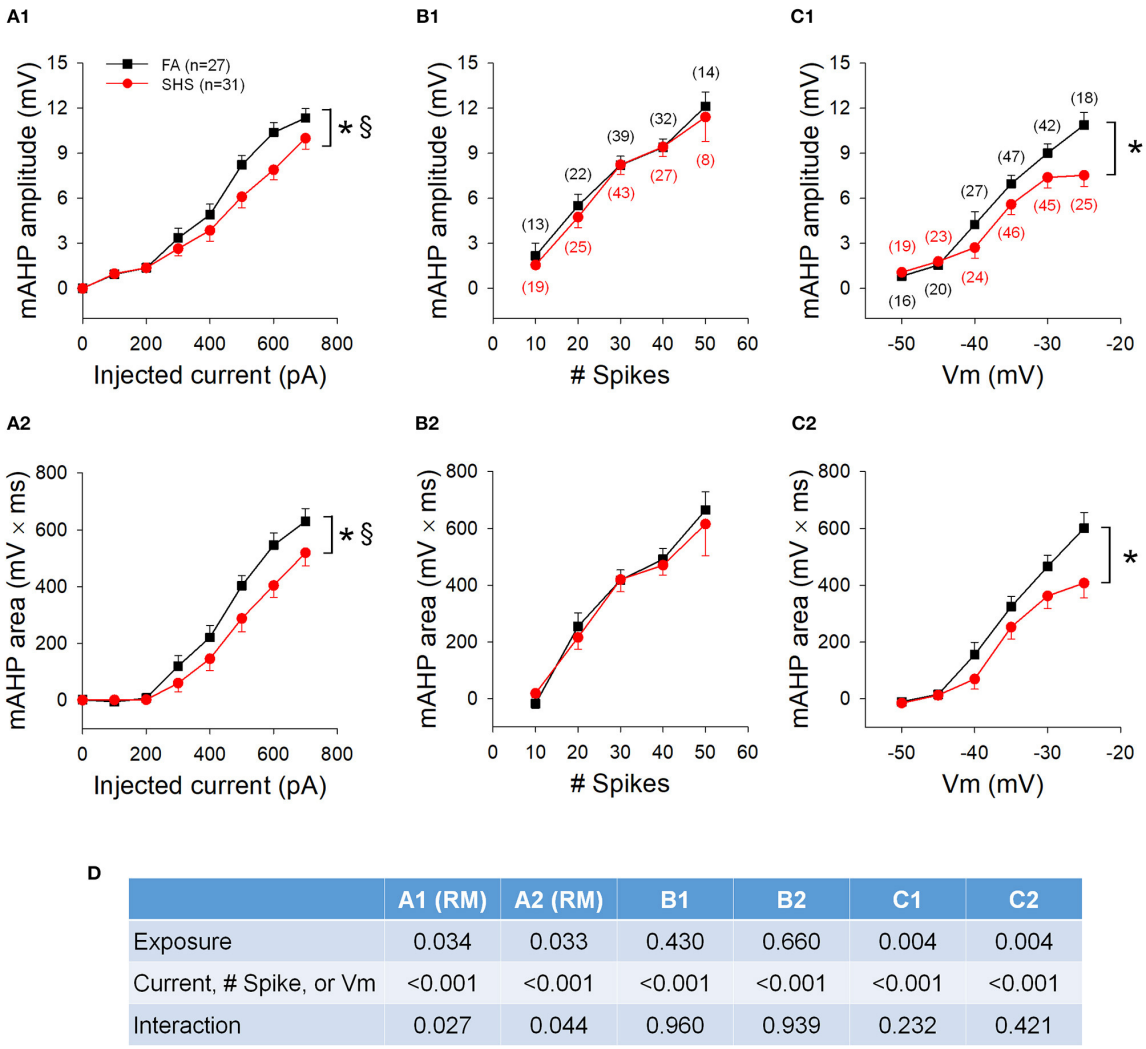
Effects of SHS on mAHP amplitude and mAHP area. **(A)** mAHP amplitude **(A1)** and area **(A2)** at the same injected currents were smaller in the SHS exposed group. **(B)** mAHP amplitude **(B1)** and area **(B2)** plotted against the total number of spikes discharged during the 1 s step current injection. There was no difference between the FA and SHS groups. **(C)** mAHP plotted against average membrane voltage (Vm) during 1 s step current injections showing smaller mAHP amplitude **(C1)** and area **(C2)** in the SHS group. **(D)** Two-way ANOVA results. RM, repeated measures; Numbers in parentheses indicate number of neurons. *p < 0.05 SHS vs. FA, ^§^*p* < 0.05 exposure x current interaction.

mAHP amplitude and area (Figure 5A), measured at the cessation of step current injections, were significantly smaller in SHS CVNs (*p* < 0.05, FA vs. SHS) and the difference was greater at higher injected currents (*p* < 0.05, interaction). At same membrane voltages, the SHS CVNs had significant smaller mAHP (Figure 5C). These data suggest a reduced SK channel activity after SHS exposure. When grouped by total spike number, these differences were no longer significant (Figure 5B), suggesting that spiking activity of CVNs is tightly linked to the magnitude of mAHP and thus, SK channel activity. ANOVA results are presented in Figure 5D.

### Spiking Response to Ramp Current Injections

**Figure 6:**
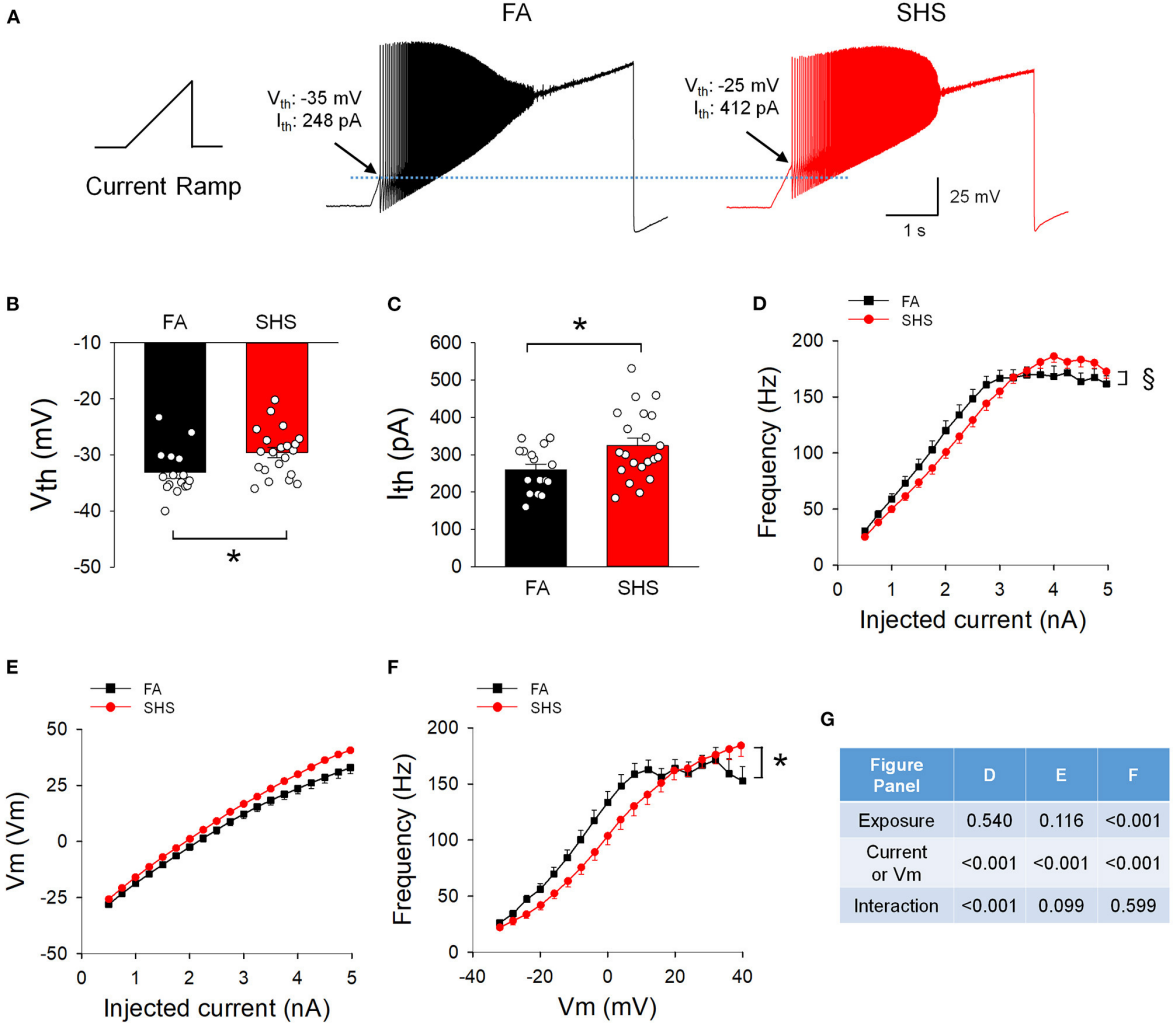
Effects of SHS on spiking responses to a ramp current injection (5 nA in 5 s). **(A)** Example traces of neurons from one FA and one SHS exposed group. Arrows indicate the membrane voltage and injected current where the first action potential was discharged and was designated as voltage threshold (Vth) and current threshold (Ith) respectively. **(B)** Group data of voltage threshold showing a significantly higher threshold for neurons from the SHS group (*t*-test, *p* = 0.0166). **(C)** Group data of current threshold showing that higher input currents were required to evoke an action potential in the SHS group (*t*-test, *p* = 0.0141). **(D)** Instantaneous frequency during the 5-s ramp current injection. The significant exposure by current interaction indicates that the SHS group had a flatter increase in instantaneous frequency as the injected current increased (flatter input-output relationship). **(E)** Membrane voltage during the ramp current injection showing no difference between FA and SHS, suggesting that the flatter input-output relation to current injection is not due to a smaller membrane voltage response to the injected current. **(F)** Instantaneous frequency plotted against membrane voltage during the 5-s ramp current injection. The SHS group had lower spiking frequency at the same voltages. **(G)** Two-way repeated measures ANOVA results for panels **(D–F)**. Sample size: *n* = 16 for FA and *n* = 21 for SHS. *p < 0.05 SHS vs. FA, ^§^*p* < 0.05 exposure x current interaction.

Figure 6A shows example spiking responses from FA and SHS neurons to ramp current injections. On average, CVNs in the SHS group had higher voltage and current thresholds (~3.5 mV and ~65 pA higher, respectively) (Figures 6B,C). While CVNs from both FA and SHS groups increased instantaneous frequency concurrent with increasing ramp currents (frequency maxed out at approximately 170 Hz, Figure 6D), SHS neurons had a flatter input-output relationship (*p* < 0.05, interaction). See ANOVA results in Figure 6G. This flatter spiking response was not the result of a more hyperpolarized membrane voltage because these SHS neurons tended (not significant) to have higher membrane voltages for the same injected current (Figure 6E). In fact, CVNs from the SHS group had significantly lower spiking frequency at most given membrane voltages (Figure 6F).

### Ramp Current Injections and AP Waveform

**Figure 7:**
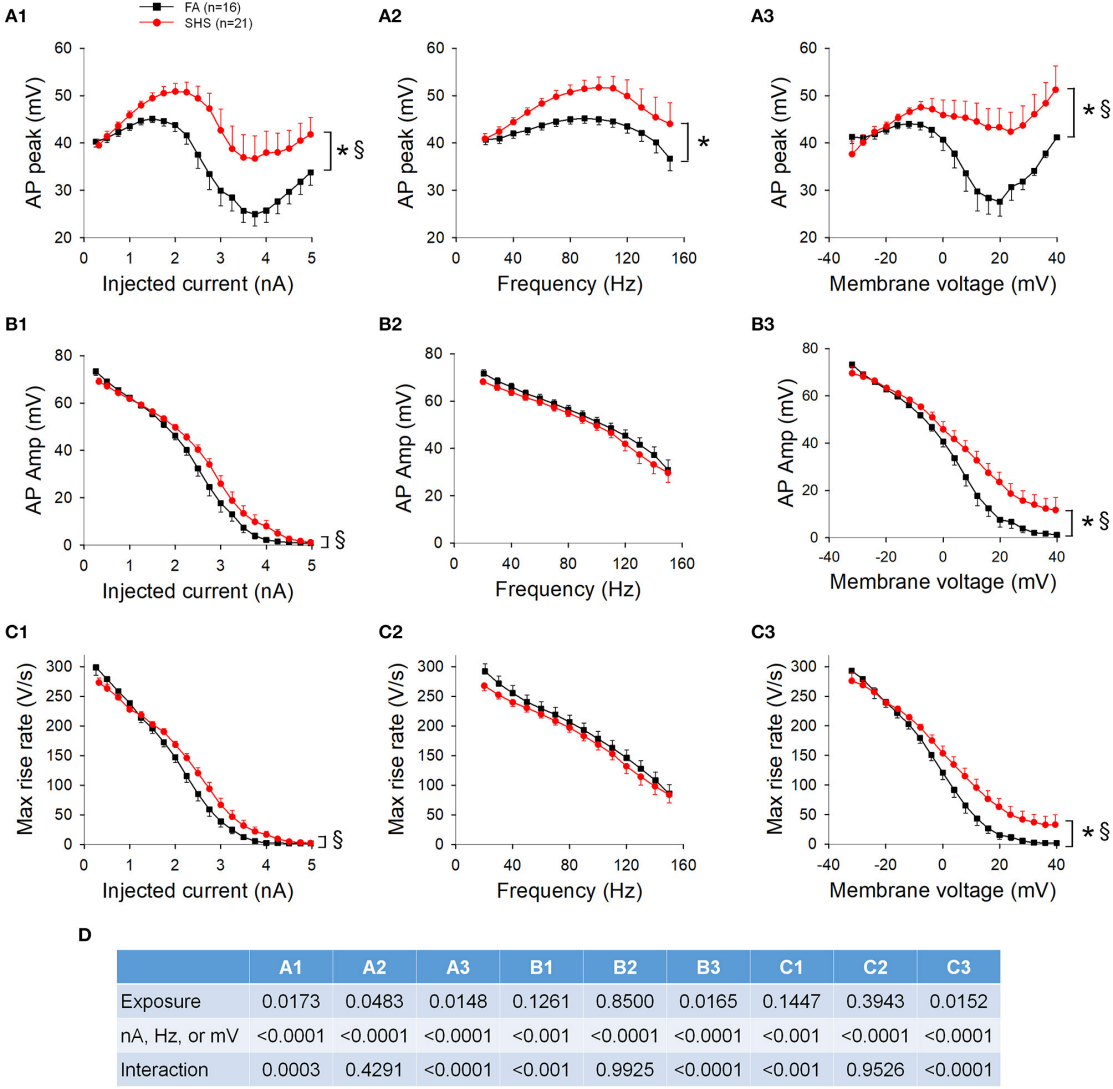
Effects of SHS on AP upstroke characteristics over the 5-s ramp current injection. **(A)** AP peak was higher in SHS group at higher injected current **(A1)**, discharge frequency **(A2)**, and membrane voltage **(A3)**. **(B)** AP amplitude was larger at higher injected current **(B1)** and membrane voltage **(B3)**. AP amplitude was similar at the same discharge frequency **(B2)**. **(C)** AP maximum rise rate. As the injected current increased, the decrease in the rise rate was steeper in the FA group **(C1)**. The difference vanished when plotting against discharge frequency **(C2)** and persisted when plotting against membrane voltage **(C3)**. **(D)** Two-way repeated measures ANOVA results. *p < 0.05 SHS vs. FA, ^§^*p* < 0.05 interaction.

AP waveform measurements were averaged three ways: (1) based on injected current at 250 pA increments, (2) based on instantaneous frequency at 10 Hz increments, and (3) based on membrane voltage at 4 mV increments. Figure 7 shows AP upstroke characteristics over a 5 s ramp current injection. As the current ramped up, AP peak increased, reaching a maximum peak of around 2 nA, which subsequently decreased due to sodium channel inactivation (Figure 7A1, also see example traces in Figure 6A). Notably, the maximum absolute value of AP peak was more depolarized in SHS neurons (~51 vs. ~45 mV, SHS vs. FA, respectively). See ANOVA results in Figure 7D. A higher AP peak was consistently observed when the data were grouped based on injected current (Figure 7A1), instantaneous frequency (Figure 7A2) and membrane voltages (Figure 7A3) (*p* < 0.05 FA vs. SHS). A significant interaction between exposure and current/membrane voltage suggested that the difference between FA and SHS was greater at higher injected current and membrane voltage. In SHS neurons, AP amplitudes were larger at higher injected currents (Figure 7B1, *p* < 0.05, interaction) and more depolarized membrane voltages (Figure 7B3, *p* < 0.05 for exposure effect and interaction). Even though the SHS group had a higher AP peak at same discharge frequency (Figure 7A2), the AP amplitude was not different (Figure 7B2). This likely reflects the higher membrane voltage needed by CVNs in the SHS group to achieve the same discharge frequency as FA CVNs (Figure 6F). Similarly, the maximum rise rate of the AP upstroke was higher in SHS neurons at higher injected currents (Figure 7C1) and membrane voltages (Figure 7C3); no difference was observed at the same instantaneous frequencies (Figure 7C2).

**Figure 8:**
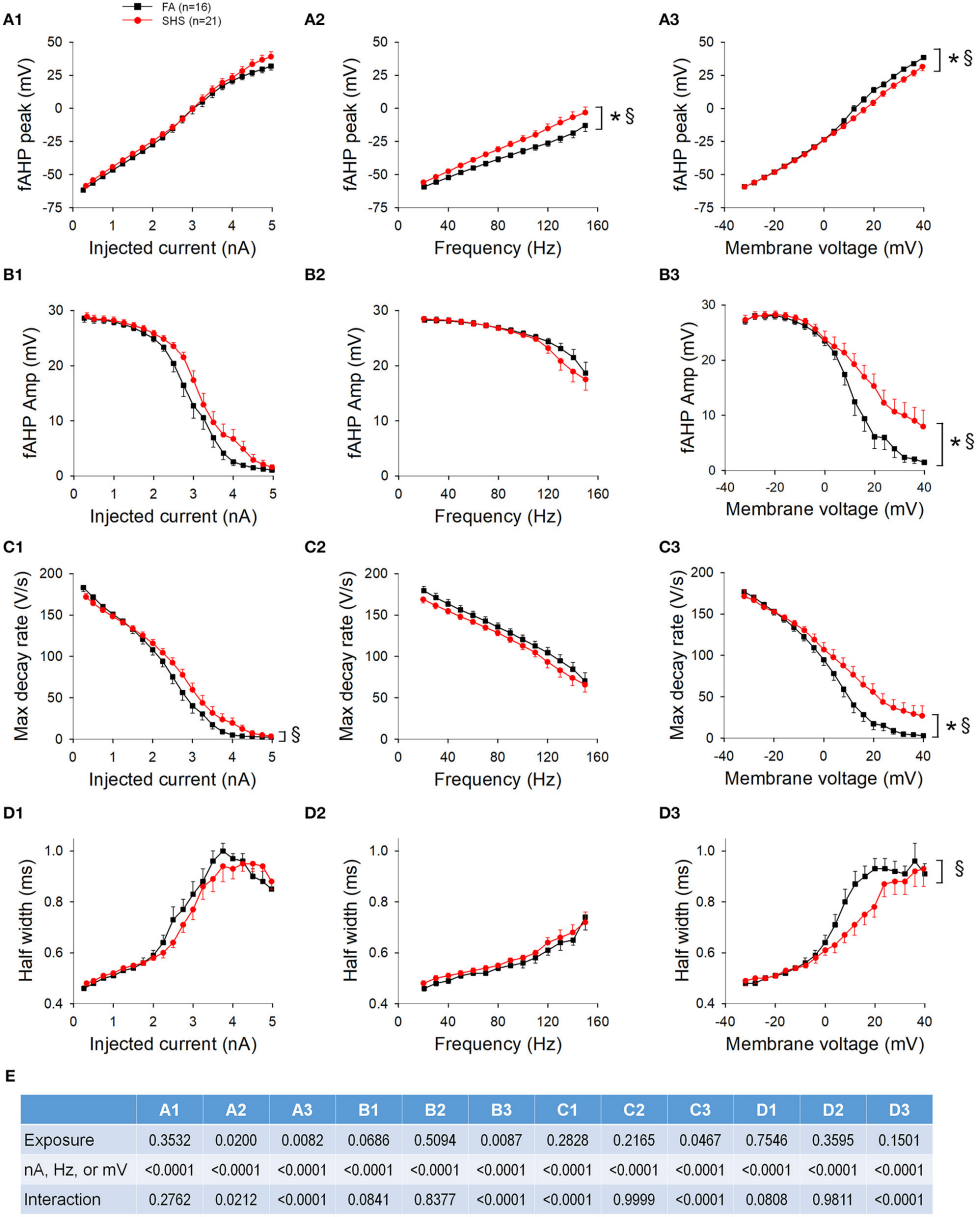
Effects of SHS on AP downstroke characteristics over the 5-s ramp current injection. **(A)** intra-train fAHP peak. There was no difference in fAHP peak when plotting against injected current **(A1)**. However, there was a significantly more depolarized fAHP peak in the SHS group at same discharge frequency **(A2)** and a small but significantly more hyperpolarized fAHP peak at positive membrane voltages **(A3)**. **(B)** fAHP amplitude in the SHS group was not different from the FA group with increasing injected current **(B1)** and discharge frequency **(B2)** but was significantly larger at higher membrane voltage **(B3)**. **(C)** AP maximum decay rate showing no difference between FA and SHS at same injected current **(C1)** and discharge frequency **(C2)** but a greater decay rate at positive membrane voltages in the SHS group **(C3)**. **(D)** AP half width was smaller in the SHS group at higher membrane voltage **(D3)**. There was no difference at the same injected currents **(D1)** or discharge frequency **(D2)**. *p < 0.05 SHS vs. FA, ^§^*p* < 0.05 interaction. **(E)** Two-way repeated measures ANOVA results.

The fAHP peak was not different between FA and SHS at the same injected current (Figure 8A1) but SHS CVNs had a more depolarized fAHP peak at same discharge frequency (Figure 8A2) and membrane voltage (Figure 8A3) (*p* < 0.05, FA vs. SHS). The exposure effects were greater at higher discharge frequency and member voltage (*p* < 0.05, interaction). fAHP amplitude was only larger in the SHS group when the data was grouped by membrane voltage (Figure 8B) and the difference was greater at higher membrane voltages (*p* < 0.05 for exposure effect and interaction). The larger fAHP amplitude was associated with a faster maximum AP decay rate (Figure 8C, *p* < 0.05 interaction) and shorter AP half width (Figure 8D) at higher member voltage (Figure 8D3, *p* < 0.05, interaction). Full ANOVA results are presented in Figure 8E.

### Effects of Apamin: Step Current Injection

**Figure 9:**
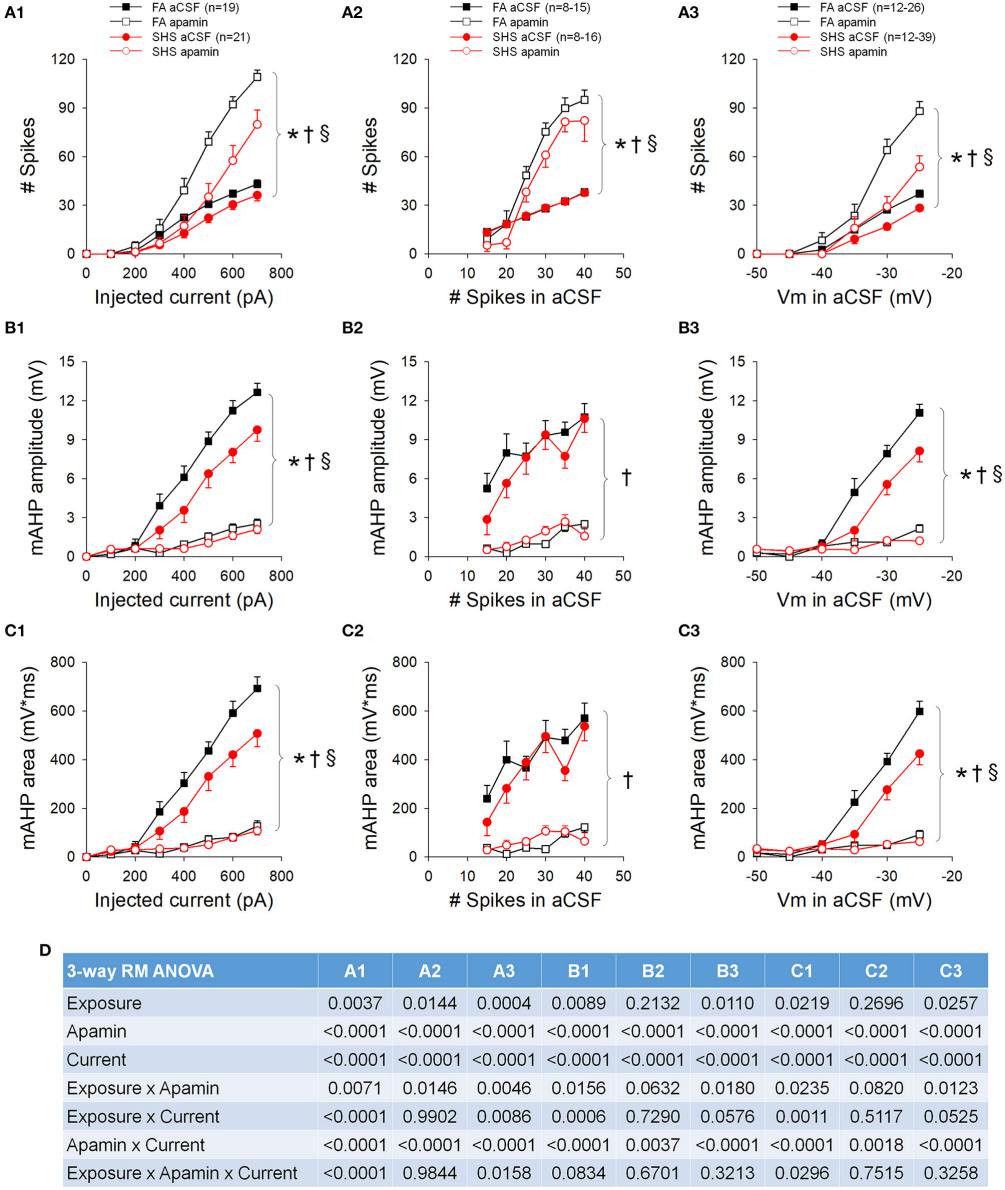
Effects of apamin on spiking responses to step current injections. **(A)** Spiking responses to step current injections before (aCSF) and during apamin perfusion. Apamin had a smaller effect in the SHS group at a given injected current **(A1)**, total number of spikes **(A2)**, and membrane voltage **(A3)**. **(B)** mAHP amplitude before and during apamin perfusion. Apamin had a smaller effect in the SHS group at same injected current **(B1)** and membrane voltage **(B3)** but not at same total discharge rate **(B2)**. **(C)** mAHP area before and during apamin perfusion. As in the case of mAHP amplitude, apamin had a smaller effect in the SHS group at same injected current **(C1)** and membrane voltage **(C3)** but not at same total discharge rate **(C2)**. **(D)** Three-way repeated measures ANOVA results. *p < 0.05 SHS vs. FA, ^†^*p* < 0.05 apamin vs. aCSF, ^§^*p* < 0.05 exposure x apamin interaction.

While perfusion with apamin significantly increased both current and voltage thresholds, this effect was greater in CVNs from the SHS group (Table 1). Blocking SK channels with apamin significantly increased neuronal spiking responses to step current injections (*p* < 0.05, apamin effect), an effect that was significantly smaller in the SHS group (*p* < 0.05, exposure x apamin interaction) (Figure 9A1). When grouping the data by pre-apamin total spike number (Figure 9A2), apamin resulted in a smaller increase in spiking response in SHS neurons at frequencies >20 Hz (*p* < 0.05, apamin effect and exposure x apamin interaction). Grouping the data by pre-apamin membrane voltages (Figure 9A3) also yielded smaller apamin-induced increases in spiking response in the SHS group (*p* < 0.05, apamin effect and exposure x apamin interaction). These data suggest a reduced SK channel activity after SHS exposure. Apamin perfusion eliminated the mAHP (Figures 9B,C), which confirms that mAHP is mediated by activation of SK channels (*p* < 0.05, apamin effect). At the same injected current (Figures 9B1,C1) and membrane voltage (Figures 9B3,C3), apamin had a smaller effect on mAHP amplitude and area on SHS CVNs (*p* < 0.05, exposure x apamin interaction), consistent with a reduced SK channel activity in SHS CVNs. Grouping the data by pre-apamin discharge frequency (Figures 9B2,C2) did not reveal any exposure-related apamin effects, which is consistent with the data (Figure 5B) showing that SHS did not affect mAHP amplitudes/areas when discharge frequencies were the same and that spiking activity is tightly coupled to SK channel activity. Full ANOVA results are presented in Figure 9D.

**Table 1:** Apamin's effects on voltage and current thresholds.

	**FA**		**SHS**		**ANOVA** ***p*****-values**		
	**aCSF**	**Apamin**	**aCSF**	**Apamin**	**Exposure**	**Drug**	**interaction**
I_min_ (pA)	305 ± 14	316 ± 21	390 ± 26^*^	433 ± 26^*^	0.001	0.061	0.249
I_th_ (pA)	241 ± 17	294 ± 27^†^	331 ± 27^*^	450 ± 36^*^^†^	0.005	<0.001	0.026
V_th_ (mV)	−34 ± 1	−32 ± 1^†^	−29 ± 1^*^	−25 ± 2^*^^†^	0.003	<0.001	0.025

*I_min_: current threshold from the step protocol; n = 19 for FA, n = 21 for SHS*.

*I_th_ and V_th_: current and voltage thresholds from the ramp protocol; n = 10 for FA, n = 13 for SHS*.

* *p < 0.05 vs. FA (Fisher's LSD)*.

† *p < 0.05 vs. aCSF (Fisher's LSD)*.

For intra-train fAHP, apamin evoked a more depolarized fAHP peak in SHS neurons at same injected currents, discharge frequencies, and membrane voltages (Supplementary Figure 2A). However, apamin had no effect on fAHP amplitude except at 31–35 discharge rate (Supplementary Figure 2B).

### Effects of Apamin: Ramp Current Injection

**Figure 10:**
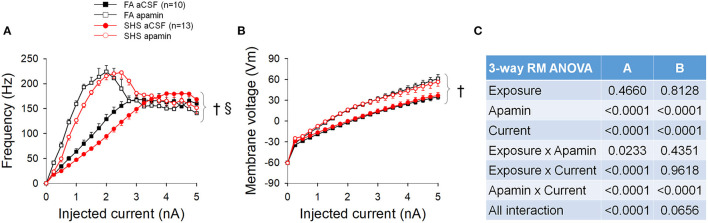
Spiking and membrane voltage responses to a ramp current injection in the absence and presence of apamin. **(A)** Apamin significantly increased spiking response to current injection. **(B)** Apamin similarly increased membrane voltage response to current injection. **(C)** Three-way repeated measures ANOVA results. ^†^*p* < 0.05 apamin vs. aCSF, ^§^*p* < 0.05 exposure x apamin interaction.

Apamin perfusion increased the spiking input-output relationship by increasing the maximum spiking frequency from ~168 to ~223 Hz (Figure 10A). A flatter input-output relationship for SHS CVNs persisted in the presence of apamin (*p* < 0.05, exposure effect). Full ANOVA results are presented in Figure 10C. Apamin also increased the membrane voltage for the same injected currents (Figure 10B, *p* < 0.05, apamin effect), suggesting that opening of SK channel during repetitive spiking shapes spiking activity by, at least in part, lowering the membrane voltage. There was no difference between FA and SHS CVNs with regards to apamin's effects on membrane voltage.

**Figure 11:**
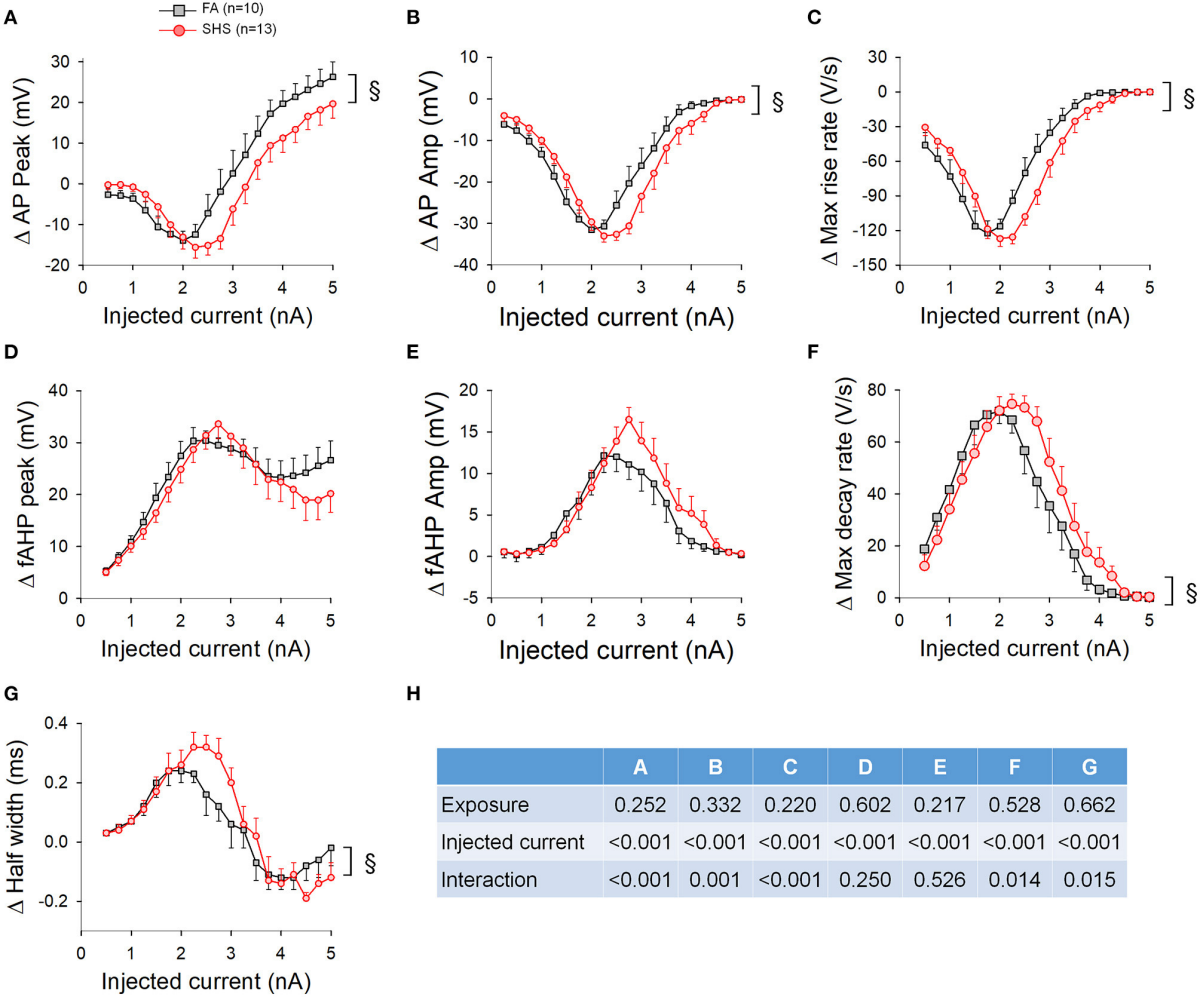
Apamin-induced changes in AP characteristics over the 5-s ramp current injection. Apamin-induced changes in AP peak **(A)**, AP amplitude **(B)**, and maximum rise rate **(C)** showed a shift toward higher injected current in the SHS group. Apamin-induced changes on fAHP peak **(D)** and amplitude **(E)** were not different between the FA and SHS groups. AP maximum decay rate **(F)** and half width **(G)** also showed a shift toward higher injected current in the SHS group. **(H)** Two-way repeated measures ANOVA results. ^§^*p* < 0.05 interaction.

With regard to apamin's effect on AP characteristics, apamin decreased AP peak, AP amplitude, and maximum rise rate (Figures 11A–C) and increased fAHP peak, fAHP amplitude, maximum decay rate, and AP half width (Figures 11D–G). Except for fAHP peak and fAHP amplitude, there was a significant interaction in the ANOVA results (Figure 11H), suggesting that apamin caused a rightward shift (toward higher injected currents) in SHS CVNs.

## Discussion

The main finding of this study is that four weeks of SHS exposure, at an environmentally relevant level, reduced intrinsic excitability of CVNs that project to the heart—a finding that is consistent with the hypothesis. While several mechanisms appear to contribute to the reduced cardiac vagal neuronal excitability, we found SHS exposure increased the voltage/current threshold required for AP generation while blunting the neuronal spiking response to depolarizing stimuli. Contrary to our hypothesis, the SHS-reduced intrinsic excitability was not mediated by an increased SK channel activity. Blocking SK channels with apamin exaggerated the difference in spiking response between FA and SHS groups, suggesting that reduced SK channel activity may be a protective response to compensate for SHS-induced reductions in cardiac vagal neuronal excitability. Furthermore, CVNs from the SHS group had higher AP and fAHP amplitudes at positive membrane voltages, raising the possibility that attenuated inactivation of voltage-gated channels underlying AP waveform might function to preserve the full range of potential output frequency. Even though the maximum output frequency did not change in SHS CVNs, higher input stimuli will be required to evoke same output responses after SHS exposure (i.e., a flatter input-output relationship)—these data may explain SHS exposure-reduced HRV.

### SHS-Induced CVNs Plasticity

Throughout the central nervous system (CNS), alterations in the expression, distribution, and properties of a whole host of ion channels underlie plasticity of neuronal excitability that manifest as changes in resting membrane potential, whole cell resistance, AP threshold, spiking frequency, spiking pattern, or input-output (stimulus-frequency) relationship. We found that SHS exposure did not alter basic membrane properties (e.g., resting membrane potential and whole cell resistance) or discharge pattern (e.g., onset delay and spiking adaptation during persistent depolarization). However, SHS exposure decreased CVNs intrinsic excitability by increasing the threshold required to generate an AP and by decreasing the neuronal stimulus-frequency (input-output) relationship. Importantly, CVNs have no spontaneous activity, meaning their output is determined by synaptic inputs (Mendelowitz, 1996; Pham et al., 2009). Thus, SHS-induced decreases in CVN excitability could serve to mute the magnitude of synaptic inputs, and thereby dampen the requisite dynamism of vagal cardiac regulation.

There are more than 7,000 chemicals in SHS (Moritsugu, 2007; Warren et al., 2014) that can be divided into three major components: nicotine, carbon monoxide, and particulate matter. Suspended particles generated from the burning cigarette may be particularly important in SHS-reduced excitability of CVNs. With regard to nicotine, contrary to our finding of a reduced excitability after SHS exposure, prenatal nicotine exposure, at a level similar to a fetus experienced in moderate to heavy smoking mom, has been shown to increase excitatory inputs to CVNs (Huang et al., 2004; Kamendi et al., 2006). Studies on the effects of carbon monoxide (increased carboxyhemoglobin level) on autonomic cardiovascular regulation of blood pressure and heart rate had yield mixed and inconsistent results (Aronow et al., 1979; Farber et al., 1990). Finally, exposure to particulate matter has been consistently shown to have the same cardiovascular consequences as SHS, including a decreased HRV (Pope and Dockery, 2006). Thus, while the SHS-reduced excitability of CVNs is unlikely to be caused by a single component, suspended particles may play an important role.

### Spiking Activity and AHP

Blocking SK channels with apapmin significantly increased spiking activity of CVNs, consistent with the role of SK channel in reducing spiking activity. We found that, at an input stimulus of 700 pA, SK currents dampened the spiking activity by ~61% in FA neurons and by ~53% in SHS neurons (Figure 9A1). Furthermore, SK channel activity appears to be tightly linked to CVN final spiking output frequency, and this relationship did not differ between FA and SHS (Figures 9B2,C2). These data suggest that SK channels play an important role in spiking activity of CVNs.

Contrary to our hypothesis, our data suggest that the SHS-reduced spiking activity in CVNs is not associated with an increased SK channel activity. In fact, application of the SK channel blocker resulted in a significantly smaller increase in spiking activity in the SHS group. Consistent with this finding is the observation that mAHP, a measurement of SK channel activity, was smaller in the SHS CVNs. These data suggest that that SHS exposure reduces SK channel activity. Because the difference in spiking response between FA and SHS was greater without SK channels, the data suggest that mechanisms other than an altered SK channel dampened CVN spiking activity and a reduced SK channel activity may serve to minimize the SHS-induced decrease in the input-output relationship.

We show that CVNs from SHS group had larger fAHP amplitudes, maximum decay rates and AP half widths at membrane voltages above 0 mV (Figure 8). These data suggest that these SHS CVNs may shift potassium channel inactivation toward a more positive voltage, minimizing the broadening of action potential waveform at membrane voltages above zero. A more compact action potential waveform may help to maintain the fidelity of signal transmission. Several potassium channels contribute to the downstroke of AP and fAHP, including voltage-gated potassium (Kv) and voltage-dependent large conductance calcium-activated potassium (BK) channels. BK currents have been implicated in AP repolarization, AP half width, and intra-train fAHP in CVNs (Lin et al., 2014). Both Kv and BK channels are good candidates for future investigation.

### AP Threshold and Upstroke

We found that CVNs from the SHS group had larger AP amplitudes and maximum rise rates at membrane voltages above 0 mV (Figures 7B3,C3). In the CNS, most of the APs are generated by activation of tetrodotoxin (TTX)-sensitive sodium currents that have fast inactivation kinetics. In addition, a slow inactivation of fast, TTX-sensitive sodium current is believed to be inherent to CNS neurons (Baranauskas, 2007). Whether the observed SHS-induced shift in AP amplitude was due to changes in the ratio of fast-to-slow inactivation of sodium channels or voltage-dependence of channel inactivation warrants further investigation.

The SHS-increased voltage threshold may also be due to changes in the sodium channel properties themselves. Changes in AP threshold that alter neuronal output have been observed in various neuronal networks associated with disorders such as chronic pain, stress, and epilepsy (Beck and Yaari, 2008). For example, in the hippocampus, long-term potentiation is associated with decreased AP threshold, presumably mediated by a change in voltage-dependence of voltage-gated sodium channels (Xu et al., 2005). In animal models of chronic pain, an increase in voltage-gated sodium currents (increased channel expression) has been shown to lower the AP threshold of dorsal root ganglion neurons (Wang et al., 2007). While the characterization of sodium channel properties is beyond the scope of this study, the higher AP threshold observed in SHS neurons suggests that SHS may impact sodium channel expression, conductance, and/or voltage-dependence.

Taken together, our data suggest that SHS induced decrease in neuronal excitability of CVNs likely involved alterations of multiple channels. Of which, changes in voltage-gated sodium channels are likely candidate to mediate the SHS-increased AP threshold and voltage-gated potassium channels (Kv and BK) may contribute to a SHS-reduced input-output relationship of CVNs.

### Current Injection Protocols

The step current injection protocol, a widely used protocol for testing neuronal intrinsic excitability, was used for spiking response to depolarization. The steps used in this study evoked spiking frequencies that fall within the previously reported discharge rate for CVNs in *in vivo* studies. The basal activity of CVNs measured *in vivo* in anesthetized animals has a discharge range of 0–9 Hz (Gold and Cohen, 1984; Jones et al., 1998; Rentero et al., 2002; O'Leary and Jones, 2003). Upon activation of pulmonary C-fibers with right atrial administration of phenybiguanide, these CVNs increased their discharge rate to 20–40 Hz (Jones et al., 1998; O'Leary and Jones, 2003). Thus, spiking response results from our step current injection protocol may reflect vagal activities both at rest and during reflex modulation.

A second current injection protocol (ramp protocol) was used to more accurately measure voltage and current threshold for AP generation. The current/membrane voltage range achieved with the ramp protocol may, at best, only be applicable to pathology conditions and only at the lower current part of the ramp. However, the ramp protocol offers a quick way to assess inactivation properties of the voltage-gated sodium and potassium channels underlying the AP. Our results suggest that SHS may alter inactivation characteristics of these voltage-gated channels.

The hyperpolarizing pre-pulse protocol used to determine the delay in spiking response to depolarization is well-established. In general, RS neurons had higher spiking activity than DS neurons. These two neuronal types have been shown, in the nucleus tractus solitarii (NTS), to receive different sensory afferent inputs and have different sensitivity to capsaicin and A-type potassium current density (Bailey et al., 2002). We previously showed, in hibernating hamsters, that as temperature decreases, RS neurons became the dominant neurons for signal transduction in the NTS (Sekizawa et al., 2012). A change in the neuronal phenotype could contribute to SHS-induced differences in neuronal excitability. However, we found no change in the ratio of RS-to-DS in CVNs after SHS exposure (Supplementary Table 1). Thus, SHS-reduced excitability is not due to a shift in neuronal phenotype.

### Perspective

SHS-reduced excitability of CVNs may underlie the reduced HRV to SHS exposure. Although 70% of deaths attributable to SHS exposure are cardiovascular in nature (Wells, 1994; Kritz et al., 1995), SHS-induced decreases in vagal regulation and autonomic imbalance may have broader implications. Autonomic imbalance is not just associated with cardiovascular disease, but with myriad CNS disorders such as Parkinson's Disease (Haapaniemi et al., 2001), Fragile X syndrome (Boccia and Roberts, 2000), and depression (Agelink et al., 2001). Furthermore, recent studies suggested that air pollution, including SHS, is linked to CNS disorders and neurodegenerative diseases (Calderon-Garciduenas et al., 2015; Vani et al., 2015; Kim et al., 2020; Shabani, 2021). Reducing SHS exposure in non-smokers with smoke-free policies in public venues could have a broader benefit to public health beyond pulmonary and cardiovascular consequences.

## Conclusion

Environmentally relevant SHS exposure can reduce neuronal excitability of CVNs. Given the strong link between air pollution (including tobacco smoke) and reduced HRV in both animal models and human cohorts, the data presented herein provide a biological basis for how neuronal impairments from SHS may impart cardiovascular consequences. SHS exposure decreased neuronal input-output relationship in CVNs, in part by increasing the threshold required for AP generation and in part by reducing the neuronal responsiveness to depolarizing stimuli. Reduced intrinsic excitability may also be associated with a shift in voltage-dependence or inactivation of the voltage-gated channels that contribute to the AP waveform. This may reflect a compensatory mechanism, as a right shift in the inactivation properties of voltage-gated channels could protect AP generation at higher membrane voltages in an effort to preserve the firing frequency range.

## Data Availability Statement

The original contributions presented in the study are included in the article/Supplementary Material, further inquiries can be directed to the corresponding author.

## Ethics Statement

The animal study was reviewed and approved by Institutional Animal Care and Use Committee at the University of California, Davis.

## Author Contributions

KP, CW, and C-YC contributed to the conception of design of the work. JS, SP, EK, Y-JC, and C-YC contributed to the acquisition, analysis, or interpretation of data for the work. All authors contributed to drafting and/or revising it critically for important intellectual content, approved publication of the content, and agreed to be accountable for all aspects ofthe work.

## Funding

This work was supported by the National Institute of Environmental Health Sciences (NIEHS) grant R01 ES025229 (to C-YC). EK was supported by Training Program in Basic & Translational Cardiovascular Science (T32 HL086350) and Advanced Training in Environmental Toxicology (T32 ES007059).

## Conflict of Interest

The authors declare that the research was conducted in the absence of any commercial or financial relationships that could be construed as a potential conflict of interest.

## Publisher's Note

All claims expressed in this article are solely those of the authors and do not necessarily represent those of their affiliated organizations, or those of the publisher, the editors and the reviewers. Any product that may be evaluated in this article, or claim that may be made by its manufacturer, is not guaranteed or endorsed by the publisher.

## References

[B1] Agelink M. W. Majewski T. Wurthmann C. Postert T. Linka T. Rotterdam S.. (2001). Autonomic neurocardiac function in patients with major depression and effects of antidepressive treatment with nefazodone. J. Affect. Disord. 62, 187–198. 10.1016/S0165-0327(99)00202-5 11223106

[B2] Aronow W. S. Stemmer E. A. Zweig S. (1979). Carbon monoxide and ventricular fibrillation threshold in normal dogs. Arch. Environ. Health 34, 184–186. 10.1080/00039896.1979.10667394 453927

[B3] Bailey T. W. Jin Y. H. Doyle M. W. Andresen M. C. (2002). Vanilloid-sensitive afferents activate neurons with prominent A-type potassium currents in nucleus tractus solitarius. J. Neurosci. 22, 8230–8237. 10.1523/JNEUROSCI.22-18-08230.2002 12223577 PMC6758120

[B4] Baranauskas G. (2007). Ionic channel function in action potential generation: current perspective. Mol. Neurobiol. 35, 129–150. 10.1007/s12035-007-8001-0 17917103

[B5] Barnoya J. Glantz S. A. (2005). Cardiovascular effects of secondhand smoke: nearly as large as smoking. Circulation 111, 2684–2698. 10.1161/CIRCULATIONAHA.104.492215 15911719

[B6] Beck H. Yaari Y. (2008). Plasticity of intrinsic neuronal properties in CNS disorders. Nat. Rev. Neurosci. 9, 357–369. 10.1038/nrn2371 18425090

[B7] Boccia M. L. Roberts J. E. (2000). Behavior and autonomic nervous system function assessed via heart period measures: the case of hyperarousal in boys with fragile X syndrome. Behav. Res. Methods Instrum. Comput. 32, 5–10. 10.3758/BF03200783 10758659

[B8] Bond C. T. Maylie J. Adelman J. P. (2005). SK channels in excitability, pacemaking and synaptic integration. Curr. Opin. Neurobiol. 15, 305–311. 10.1016/j.conb.2005.05.001 15922588

[B9] Calderon-Garciduenas L. Calderon-Garciduenas A. Torres-Jardon R. Avila-Ramirez J. Kulesza R. J. Angiulli A. D. (2015). Air pollution and your brain: what do you need to know right now. Prim. Health Care Res. Dev. 16, 329–345. 10.1017/S146342361400036X 25256239

[B10] Chen C. Y. Chow D. Chiamvimonvat N. Glatter K. A. Li N. He Y.. (2008). Short-term secondhand smoke exposure decreases heart rate variability and increases arrhythmia susceptibility in mice. Am. J. Physiol. Heart Circ. Physiol. 295, H632–639. 10.1152/ajpheart.91535.2007 18552155 PMC2519230

[B11] Cheng Z. Zhang H. Yu J. Wurster R. D. Gozal D. (2004). Attenuation of baroreflex sensitivity after domoic acid lesion of the nucleus ambiguus of rats. J. Appl. Physiol. (1985) 96, 1137–1145. 10.1152/japplphysiol.00391.2003 14617524

[B12] Corbett E. K. Batten T. F. Kaye J. C. Deuchars J. McWilliam P. N. (1999). Labelling of rat vagal preganglionic neurones by carbocyanine dye DiI applied to the heart. Neuroreport 10, 1177–1181. 10.1097/00001756-199904260-00004 10363920

[B13] Farber J. P. Schwartz P. J. Vanoli E. Stramba-Badiale M. De Ferrari G. M. (1990). Carbon monoxide and lethal arrhythmias. Res. Rep. Health Eff. Inst. 1–17. discussion: 19–27. 2092724

[B14] Franklin B. A. Brook R. Arden Pope C. 3rd. (2015). Air pollution and cardiovascular disease. Curr. Probl. Cardiol. 40, 207–238. 10.1016/j.cpcardiol.2015.01.003 25882781

[B15] Gold M. R. Cohen D. H. (1984). The discharge characteristics of vagal cardiac neurons during classically conditioned heart rate change. J. Neurosci. 4, 2963–2971. 10.1523/JNEUROSCI.04-12-02963.1984 6502215 PMC6564866

[B16] Haapaniemi T. H. Pursiainen V. Korpelainen J. T. Huikuri H. V. Sotaniemi K. A. Myllyla V. V. (2001). Ambulatory ECG and analysis of heart rate variability in Parkinson's disease. J. Neurol. Neurosurg. Psychiatry 70, 305–310. 10.1136/jnnp.70.3.305 11181850 PMC1737270

[B17] Hamad S. H. Johnson N. M. Tefft M. E. Brinkman M. C. Gordon S. M. Clark P. I.. (2017). Little cigars vs 3R4F cigarette: physical properties and HPHC yields. Tob. Regul. Sci. 3, 459–478. 10.18001/TRS.3.4.7 29911130 PMC5998811

[B18] Hausberg M. Mark A. L. Winniford M. D. Brown R. E. Somers V. K. (1997). Sympathetic and vascular effects of short-term passive smoke exposure in healthy nonsmokers. Circulation 96, 282–287. 9236446

[B19] Huang Z. G. Wang X. Evans C. Gold A. Bouairi E. Mendelowitz D. (2004). Prenatal nicotine exposure alters the types of nicotinic receptors that facilitate excitatory inputs to cardiac vagal neurons. J. Neurophysiol. 92, 2548–2554. 10.1152/jn.00500.2004 15212427

[B20] Jones J. F. Wang Y. Jordan D. (1998). Activity of C fibre cardiac vagal efferents in anaesthetized cats and rats. J. Physiol. 507(Pt 3), 869–880. 10.1111/j.1469-7793.1998.869bs.x 9508846 PMC2230810

[B21] Kamendi H. Stephens C. Dergacheva O. Wang X. Huang Z. G. Bouairi E.. (2006). Prenatal nicotine exposure alters the nicotinic receptor subtypes that modulate excitation of parasympathetic cardiac neurons in the nucleus ambiguus from primarily alpha3beta2 and/or alpha6betaX to alpha3beta4. Neuropharmacology 51, 60–66. 10.1016/j.neuropharm.2006.03.001 16690087

[B22] Kim H. Kim W. H. Kim Y. Y. Park H. Y. (2020). Air pollution and central nervous system disease: a review of the impact of fine particulate matter on neurological disorders. Front. Public Health 8:575330. 10.3389/fpubh.2020.575330 33392129 PMC7772244

[B23] Kritz H. Schmid P. Sinzinger H. (1995). Passive smoking and cardiovascular risk. Arch. Intern. Med. 155, 1942–1948. 10.1001/archinte.1995.00430180034005 7575047

[B24] Lin M. Chen Q. H. Wurster R. D. Hatcher J. T. Liu Y. Q. Li L.. (2010a). Maternal diabetes increases small conductance Ca2+-activated K+ (SK) currents that alter action potential properties and excitability of cardiac motoneurons in the nucleus ambiguus. J. Neurophysiol. 104, 2125–2138. 10.1152/jn.00671.2009 20668269 PMC2957455

[B25] Lin M. Hatcher J. T. Chen Q. H. Wurster R. D. Cheng Z. J. (2010b). Small conductance Ca2+-activated K+ channels regulate firing properties and excitability in parasympathetic cardiac motoneurons in the nucleus ambiguus. Am. J. Physiol. Cell Physiol. 299, C1285–1298. 10.1152/ajpcell.00134.2010 20739619 PMC3774095

[B26] Lin M. Hatcher J. T. Chen Q. H. Wurster R. D. Li L. Cheng Z. J. (2011). Maternal diabetes increases large conductance Ca2+-activated K+ outward currents that alter action potential properties but do not contribute to attenuated excitability of parasympathetic cardiac motoneurons in the nucleus ambiguus of neonatal mice. Am. J. Physiol. Regul. Integr. Comp. Physiol. 300, R1070–1078. 10.1152/ajpregu.00470.2010 21248308 PMC3094040

[B27] Lin M. Hatcher J. T. Wurster R. D. Chen Q. H. Cheng Z. J. (2014). Characteristics of single large-conductance Ca2+-activated K+ channels and their regulation of action potentials and excitability in parasympathetic cardiac motoneurons in the nucleus ambiguus. Am. J. Physiol. Cell Physiol. 306, C152–166. 10.1152/ajpcell.00423.2012 24196530 PMC3919986

[B28] Liu R. L. Yang Y. Travers M. J. Fong G. T. O'Connor R. J. Hyland A.. (2011). A cross-sectional study on levels of secondhand smoke in restaurants and bars in five cities in China. Tob. Control 20, 397–402. 10.1136/tc.2009.033233 19748882

[B29] Mendelowitz D. (1996). Firing properties of identified parasympathetic cardiac neurons in nucleus ambiguus. Am. J. Physiol. 271(6 Pt 2), H2609–2614. 10.1152/ajpheart.1996.271.6.H2609 8997322

[B30] Mendelowitz D. (1999). Advances in parasympathetic control of heart rate and cardiac function. News Physiol. Sci. 14, 155–161. 10.1152/physiologyonline.1999.14.4.155 11390842

[B31] Moritsugu K. P. (2007). The 2006 report of the surgeon general: the health consequences of involuntary exposure to tobacco smoke. Am. J. Prev. Med. 32, 542–543. 10.1016/j.amepre.2007.02.026 17533072

[B32] O'Leary D. M. Jones J. F. (2003). Discharge patterns of preganglionic neurones with axons in a cardiac vagal branch in the rat. Exp. Physiol. 88, 711–723. 10.1113/eph8802590 14603369

[B33] Pacheco S. A. Aguiar F. Ruivo P. Proenca M. C. Sekera M. Penque D.. (2012). Occupational exposure to environmental tobacco smoke: a study in Lisbon restaurants. J. Toxicol. Environ. Health A. 75, 857–866. 10.1080/15287394.2012.690690 22788372

[B34] Peterson D. F. Coote J. H. Gilbey M. P. Futuro-Neto H. A. (1983). Differential pattern of sympathetic outflow during upper airway stimulation with smoke. Am. J. Physiol. 245, R433–437. 10.1152/ajpregu.1983.245.3.R433 6614213

[B35] Pham H. Bonham A. C. Pinkerton K. E. Chen C. Y. (2009). Central neuroplasticity and decreased heart rate variability after particulate matter exposure in mice. Environ. Health Perspect. 117, 1448–1453. 10.1289/ehp.0900674 19750112 PMC2737024

[B36] Pope C. A. 3rd Dockery D. W. (2006). Health effects of fine particulate air pollution: lines that connect. J. Air Waste Manag. Assoc. 56, 709–742. 10.1080/10473289.2006.10464485 16805397

[B37] Pope C. A. 3rd Eatough D. J. Gold D. R. Pang Y. Nielsen K. R. Nath P.. (2001). Acute exposure to environmental tobacco smoke and heart rate variability. Environ. Health Perspect. 109, 711–716. 10.1289/ehp.01109711 11485870 PMC1240375

[B38] Rentero N. Cividjian A. Trevaks D. Pequignot J. M. Quintin L. McAllen R. M. (2002). Activity patterns of cardiac vagal motoneurons in rat nucleus ambiguus. Am. J. Physiol. Regul. Integr. Comp. Physiol. 283, R1327–1334. 10.1152/ajpregu.00271.2002 12388471

[B39] Sekizawa S. Chen C. Y. Bechtold A. G. Tabor J. M. Bric J. M. Pinkerton K. E.. (2008). Extended secondhand tobacco smoke exposure induces plasticity in nucleus tractus solitarius second-order lung afferent neurons in young guinea pigs. Eur. J. Neurosci. 28, 771–781. 10.1111/j.1460-9568.2008.06378.x 18657181

[B40] Sekizawa S. Horowitz J. M. Horwitz B. A. Chen C. Y. (2012). Realignment of signal processing within a sensory brainstem nucleus as brain temperature declines in the Syrian hamster, a hibernating species. J. Comp. Physiol. A. Neuroethol. Sens. Neural Behav. Physiol. 198, 267–282. 10.1007/s00359-011-0706-x 22262373 PMC4016980

[B41] Semple S. Maccalman L. Naji A. A. Dempsey S. Hilton S. Miller B. G.. (2007). Bar workers' exposure to second-hand smoke: the effect of Scottish smoke-free legislation on occupational exposure. Ann. Occup. Hyg. 51, 571–580. 10.1093/annhyg/mem044 17846033

[B42] Shabani S. (2021). A mechanistic view on the neurotoxic effects of air pollution on central nervous system: risk for autism and neurodegenerative diseases. Environ. Sci. Pollut. Res. Int. 28, 6349–6373. 10.1007/s11356-020-11620-3 33398761

[B43] Task_Force (1996). Heart rate variability: standards of measurement, physiological interpretation and clinical use. Task force of the european society of cardiology and the North American Society of pacing and electrophysiology. Circulation 93, 1043–1065. 8598068

[B44] Vani G. Anbarasi K. Shyamaladevi C. S. (2015). Bacoside A: role in cigarette smoking induced changes in brain. Evid. Based. Complement Alternat. Med. 2015:286137. 10.1155/2015/286137 26413118 PMC4564636

[B45] Wang J. G. Strong J. A. Xie W. Zhang J. M. (2007). Local inflammation in rat dorsal root ganglion alters excitability and ion currents in small-diameter sensory neurons. Anesthesiology 107, 322–332. 10.1097/01.anes.0000270761.99469.a7 17667578 PMC1945168

[B46] Wang Z. Wang L. Tapa S. Pinkerton K. E. Chen C. Y. Ripplinger C. M. (2018). Exposure to secondhand smoke and arrhythmogenic cardiac alternans in a mouse model. Environ. Health Perspect. 126:127001. 10.1289/EHP3664 30675795 PMC6371715

[B47] Warren G. W. Alberg A. J. Kraft A. S. Cummings K. M. (2014). The 2014 surgeon general's report: “the health consequences of smoking−50 years of progress”: a paradigm shift in cancer care. Cancer 120, 1914–1916. 10.1002/cncr.28695 24687615 PMC5928784

[B48] Wells A. J. (1994). Passive smoking as a cause of heart disease. J. Am. Coll. Cardiol. 24, 546–554. 10.1016/0735-1097(94)90315-8 8034894

[B49] Wilson M. D. McGlothlin J. D. Rosenthal F. S. Black D. R. Zimmerman N. J. Bridges C. D. (2010). Ergonomics. The effect of occupational exposure to environmental tobacco smoke on the heart rate variability of bar and restaurant workers. J. Occup. Environ. Hyg. 7, D44–D49. 10.1080/15459624.2010.483980 20473817

[B50] Xu J. Kang N. Jiang L. Nedergaard M. Kang J. (2005). Activity-dependent long-term potentiation of intrinsic excitability in hippocampal CA1 pyramidal neurons. J. Neurosci. 25, 1750–1760. 10.1523/JNEUROSCI.4217-04.2005 15716411 PMC6725941

[B51] Zhang J. Fang S. C. Mittleman M. A. Christiani D. C. Cavallari J. M. (2013). Secondhand tobacco smoke exposure and heart rate variability and inflammation among non-smoking construction workers: a repeated measures study. Environ. Health 12:83. 10.1186/1476-069X-12-83 24083379 PMC3906998

