# Supplementary material for: Secondhand Smoke Decreased Excitability and Altered Action Potential Characteristics of Cardiac Vagal Neurons in Mice
Source: Front Physiol. 2021 Sep 24;12:727000. doi: 10.3389/fphys.2021.727000 (PMC8498211; doi:10.3389/fphys.2021.727000)
Supplement: Supplementary file 1 [file Data_Sheet_1.PDF]

Supplementary Material

1 Supplementary Figures and Tables

1.1 Supplementary Tables

Table 1. Membrane properties of cardiac vagal neurons after four weeks of exposure.

	Filtered Air	Secondhand Smoke
n (total)	27	31*
n (RS/DS)	17/10	16/14
Rem (mV)	-67±1	-66±1
Cm (pF)	55±2	55±2
Rm (MΩ)	112±5	105±4

*One cell was not tested for spiking phenotype.
RS: rapid-onset spiking phenotype
DS: delayed-onset spiking phenotype
Rem: resting membrane potential
Cm: membrane capacitance
Rm: whole cell membrane resistance

1.2 Supplementary Figures:

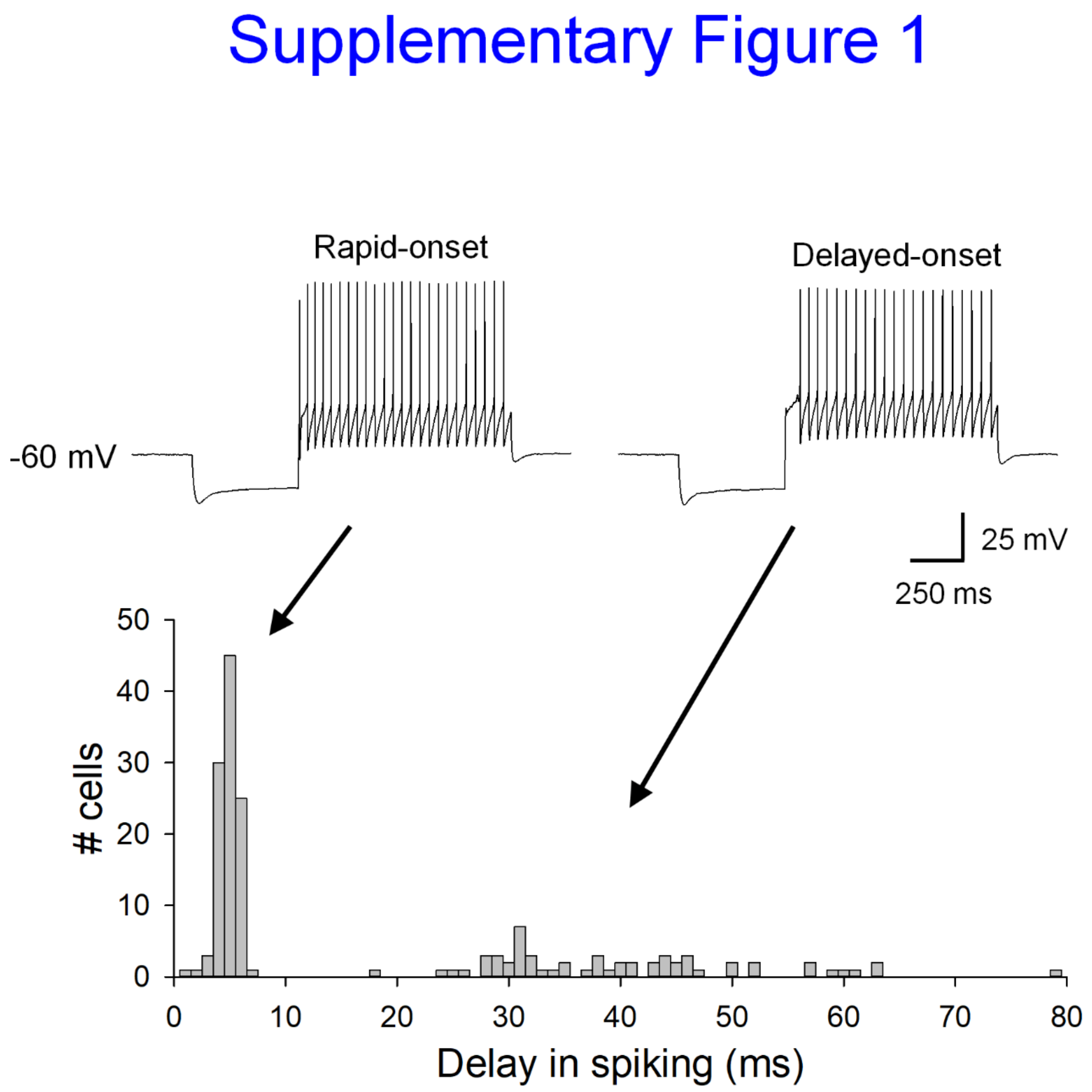

Supplementary Figure 1. Classification of neurons as a rapid-onset or delayed-onset spiking phenotype. Top, example traces of a rapid-onset and a delayed-onset neuron. Neurons were initially held at -60 mV. A pre-test hyperpolarizing current step (0.5s) was applied to hyperpolarize membrane potential to -80 mV, followed by a depolarizing current step (600 pA, 1s) to evoke action potential. Bottom, frequency histogram of delay in onset of spiking response. Neurons with spiking onset of <10 ms were classified as rapid-onset neurons and those with onset of >10 ms were classified as delayed-onset neurons.

Supplementary Figure 2

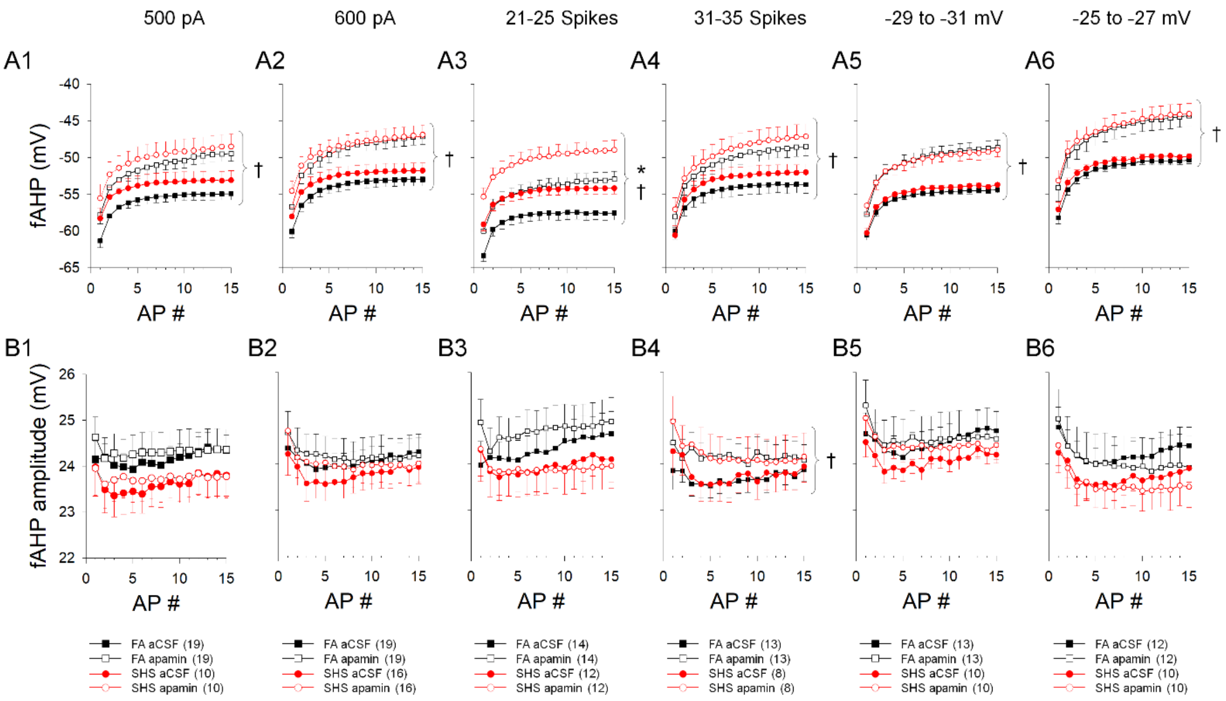

C

3-way RM ANOVA	A1	A2	A3	A4	A5	A6	B1	B2	B3	B4	B5	B6
Exposure	0.2749	0.4709	0.0099	0.4794	0.7819	0.6532	0.3365	0.6610	0.3570	0.9174	0.6180	0.4107
Apamin	<0.0001	<0.0001	<0.0001	<0.0001	<0.0001	<0.0001	0.5770	0.3777	0.4093	0.0095	0.4149	0.5865
AP#	<0.0001	<0.0001	<0.0001	<0.0001	<0.0001	<0.0001	<0.0001	<0.0001	<0.0001	<0.0001	<0.0001	<0.0001
Exposure x Apamin	0.6406	0.5403	0.4180	0.9123	0.6033	0.8820	0.9552	0.7435	0.2824	0.8236	0.6533	0.9995
Exposure x AP#	0.0040	<0.0001	0.2342	0.0487	0.9292	0.9780	0.9924	0.9794	0.0078	0.5183	0.9895	0.9995
Apamin x AP#	<0.0001	<0.0001	<0.0001	<0.0001	<0.0001	<0.0001	<0.0001	<0.0001	<0.0001	<0.0001	<0.0001	<0.0001
Exposure x Apamin x Current	0.9982	0.1380	0.8387	0.0002	0.0006	>0.9999	0.1904	0.9851	0.1362	0.9672	0.6908	0.9313

Supplementary Figure 2. Effects of apamin on intra-train fAHP. fAHP peak of the first 15 action potentials from 500 pA (**A1**), 600 pA (**A2**), 21-25 total spikes (**A3**), 31-35 total spikes (**A4**), -29 to -31 membrane voltage (**A5**), and -25 to -27 mV membrane voltage (**A6**). fAHP peak was significantly more depolarized in the presence of apamin. **B.** fAHP amplitude of the first 15 action potentials. Apamin had no significant effect on fAHP amplitude. **C.** Three-way repeated measures ANOVA results. AP #, Action Potential number. Numbers in parentheses indicate sample sizes. *p<0.05 SHS vs. FA; †p<0.05 apamin vs. aCSF.
